# Do Dietary Supplements Affect Inflammation, Oxidative Stress, and Antioxidant Status in Adults with Hypothyroidism or Hashimoto’s Disease?—A Systematic Review of Controlled Trials

**DOI:** 10.3390/antiox12101798

**Published:** 2023-09-24

**Authors:** Katarzyna Kubiak, Maria Karolina Szmidt, Joanna Kaluza, Agnieszka Zylka, Ewa Sicinska

**Affiliations:** 1 Department of Human Nutrition, Institute of Human Nutrition Sciences, Warsaw University of Life Sciences (WULS-SGGW), Nowoursynowska 166, 02-787 Warsaw, Poland; maria_szmidt@sggw.edu.pl (M.K.S.); ewa_sicinska@sggw.edu.pl (E.S.); 2 Department of Oncological Endocrinology and Nuclear Medicine, Maria Sklodowska-Curie National Research Institute of Oncology, 02-781 Warsaw, Poland; agnieszka.zylka@pib-nio.pl

**Keywords:** hypothyroidism, Hashimoto’s disease, dietary supplements, inflammation, oxidative stress, antioxidant status

## Abstract

This systematic review aims to summarise the results of controlled trials on dietary supplements (DS) usage and inflammation, oxidative stress, antioxidant status, and thyroid parameter improvement in hypothyroidism (HT)/Hashimoto’s thyroiditis (AIT) patients. The study protocol was registered with PROSPERO (no. CRD42022365149). A comprehensive search of the PubMed, Scopus, and Web of Science databases resulted in the identification of nineteen randomised controlled trials and three non-randomised studies for the review; three studies examined the effect of supplementation with vitamin D, twelve studies—with selenium, and seven studies—with other DS. Based on very limited evidence, the lack of influence of vitamin D supplementation on inflammatory parameters was found, while no studies have examined oxidative stress and antioxidant status parameters, and only one provided results for a single thyroid parameter after an intervention. Some evidence was found proving that selenium supplementation may decrease inflammation and improve thyroid parameters, but reaching a conclusion about its influence on oxidative stress and antioxidant status is not possible because of the insufficient number of studies. Additionally, due to examining other DS (e.g., multicomponent, *Nigella sativa*, and genistein) only in single studies, conclusions cannot be drawn. Further long-term, high-quality randomised controlled trials are necessary to better understand the influence of DS on inflammation, oxidative stress, and antioxidant status, as well as their potential to improve thyroid gland function in HT/AIT patients.

## 1. Introduction

Hypothyroidism (HT) (ICD-11, code 5A00) [1] is a condition of thyroid hormone deficiency [2]. In Europe, the total prevalence of diagnosed disease is 5% [3], while undiagnosed HT affects approximately an additional 4.7% of the population [4]. The prevalence of HT tends to be higher in females, individuals aged 65 years or older, and patients with autoimmune diseases [4]. HT can be overt as well as subclinical and is diagnosed based on elevated thyroid-stimulating hormone (TSH) serum concentration and decreased free thyroxine (fT4) levels [5].

Autoimmune thyroiditis (AIT), also known as Hashimoto’s thyroiditis (ICD-11, code 5A03.2) [2], is an organ-specific autoimmune disease with a prevalence of 7.5% in the adult population [6]. It is characterised by the development of autoantibodies to thyroid-specific antigens and inflammatory cell infiltration of the thyroid gland. This process ultimately results in the progressive, gradual destruction of the thyroid follicles, which subsequently leads to the frequent occurrence of HT as a consequence [7]. In the pathogenesis of AIT, genetic factors contribute approximately 70–80%, while environmental and lifestyle factors—such as excessive alcohol consumption, unbalanced exercise, obesity, poor sleep quality, and exposure to psychosocial stress—account for 20–30% [8,9]. These lifestyle factors can increase the production of reactive oxygen species and contribute to elevated thyroid inflammation [8]. 

Both AIT and HT are associated with low-grade systemic inflammation and local inflammation in the thyroid gland. In AIT, T helper (Th) cells produce cytokines, which induce thyrocytes to express surface Human Leukocyte Antigen DR (HLA-DR) and, as a result, make them susceptible to immune attack. Different Th cell subtypes secrete various inflammatory cytokines: Th1 cells secrete interferon-γ (IFN-γ), interleukin-2 (IL-2), and tumour necrosis factor-alpha (TNF-α); Th2 cells secrete IL-4 and IL-5; and Th17 cells (present in the autoimmune disease pathophysiology) secrete IL-17 and IL-23 [10]. Moreover, inflammation may be involved in the pathogenesis of different complications associated with AIT, e.g., atherosclerotic or impaired endothelial function. Results of a meta-analysis concerning the impact of HT on low-grade systematic inflammation indicated that overt HT was associated with a significant increase in C-reactive protein (CRP) levels, but levothyroxine replacement therapy decreased CRP levels [11]. Additionally, interleukins may be involved in HT—some researchers have indicated increased levels of some members of the IL-1 [12,13], IL-6 [14], IL-17 [13,15], and IL-23 families [13,15,16]; however, some of these studies did not indicate differences in the serum interleukin concentrations between AIT patients and healthy subjects [10,17]. 

By acting on the metabolism, triiodothyronine (T3) and thyroxine (T4) probably increase antioxidant potential [18]. Consequently, an increase in oxidative stress parameters can be observed in AIT patients, while antioxidant parameters decrease [19]. On the one hand, both HT and AIT patients often suffer from nutritional deficiencies [20]; on the other hand, the use of dietary supplements (DS)— recognised as a health-promoting behaviour—may offer an easy and prompt method of supplementation [21]. However, it is important to establish whether the use of DS has a positive impact on health, including thyroid parameters, inflammation and oxidative stress reduction, and an improvement in antioxidant status. To find the answer, researchers are conducting controlled trials to assess the effect of DS usage on these parameters, but their results are inconsistent. Thus, summarising their findings seems to be crucial to drawing conclusions.

Taking the above into account, the authors conducted a comprehensive systematic review to summarise the available study results regarding DS usage in relation to inflammation, oxidative stress, antioxidant status, and improvement of thyroid parameters in HT or/and AIT patients. 

## 2. Materials and Methods

This systematic review has been registered at the protocol stage in the International Prospective Register of Systematic Reviews PROSPERO, record number CRD42022365149. 

### 2.1. Literature Search

A systematic review of the literature was conducted in June 2023, following the Preferred Reporting Items for Systematic Reviews and Meta-Analyses (PRISMA) guidelines [22]. Two independent authors (K.K. and M.K.S.) have searched three databases separately: PubMed, Scopus, and Web of Science, without language restrictions. The search strategy is shown in Table S1 and was defined by combining terms related to ‘hypothyroidism’, ‘Hashimoto’s thyroiditis’, ‘dietary supplements’, ‘oxidative stress’, and ‘inflammation’. A manual search of further potentially eligible studies was performed, including references to the retrieved articles.

### 2.2. Inclusion and Exclusion Criteria

The inclusion criteria were as follows: (1) an intervention-controlled study design; (2) adults (≥18 years old); (3) hypothyroidism or Hashimoto’s thyroiditis; (4) patients in the intervention group received DS at a specified dose; (5) participants in the control group received placebo or adjuvant treatment; (6) studies where the assessment of inflammatory markers, oxidative stress, and/or antioxidant status was performed before and after the trial; and (7) articles written in English.

The exclusion criteria consisted of: (1) a study design different than an intervention; (2) participants aged under 18 years; (3) diseases other than hypothyroidism or Hashimoto’s thyroiditis of the thyroid gland; (4) pregnancy or lactation; (5) patients treated with iodine-131 (I-131); (6) patients qualified for thyroid surgery or after thyroidectomy; (7) studies where inflammation, oxidative stress, or antioxidant status parameters were not analysed; and (8) the language of the study other than English.

### 2.3. Study Selection and Data Extraction

All the identified studies were screened by two independent researchers (K.K. and M.K.S.) for eligibility. Initially, titles and abstracts were evaluated, and then a full-text evaluation was performed, taking into account the listed criteria. In the case of non-compliance, the eligibility of the article was discussed and resolved by the decision of the senior author (E.S.).

The following data were extracted from the studies as characteristics: name of the author(s), year of publication, study location, study design, number of participants in an intervention and control group, inclusion and exclusion criteria for both groups, age of participants, and biomarkers of interest. Furthermore, the information necessary in terms of results was: DS (dose and form), study duration baseline, and end-of-study biological parameter measurements for both the intervention and control groups; *p*-values within groups before and after the intervention; as well as between the intervention and control groups. If the necessary data were missing in the articles, the authors were contacted to obtain them.

### 2.4. Quality Assessment

The quality of randomised studies was assessed using the Critical Appraisal Skills Programme (CASP) Randomised Controlled Trial Standard Checklist [23]. Categorization for randomised controlled trials (RCT) was adapted from Pollock et al. [24]. Trials where most items in the tool were assessed as ‘yes’ were considered to be those with no or few limitations; trials where most items were assessed as ‘yes’ or ‘cannot tell’ were considered to be those with minor limitations; and trials with one or more questions assessed as ‘no’ were considered to be those with major limitations. Non-randomised studies were assessed using the Risk of Bias in Non-randomised Studies of Interventions (ROBINS-I) tool [25] recommended by the Cochrane Collaboration [25]. The assessment of the risk of bias in non-randomised studies was based on the ROBINS-I materials provided [25].

## 3. Results

### 3.1. Trial Selection

**Figure 1:**
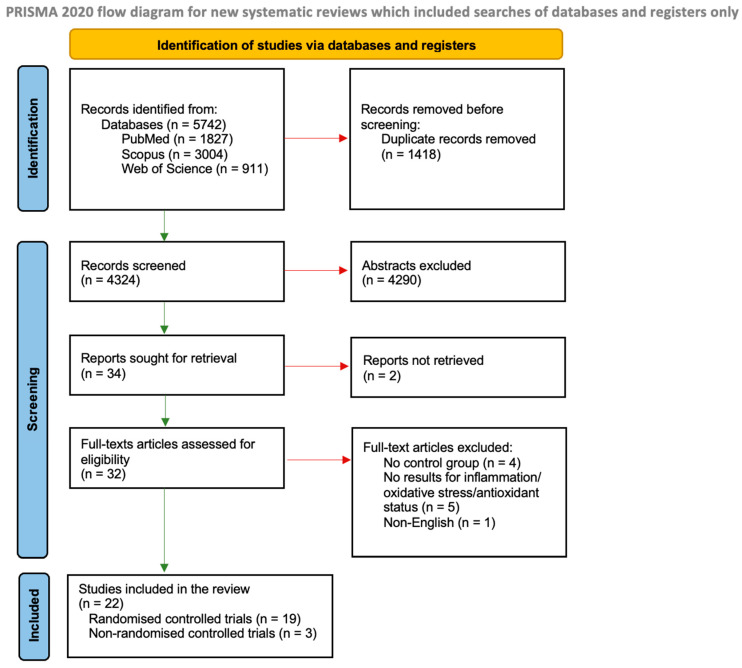
Literature review flow diagram of the selection publication process according to the Preferred Reporting Items for Systematic Reviews and Meta-Analyses (PRISMA) Statement [22].

We identified 5742 studies through the database searches, and 1418 were duplicates (Figure 1). From 4324 records, we excluded 4290 based on abstracts and two due to the lack of access to the full-text version. A total of 32 full-text articles from the databases were assessed for eligibility. A total of twenty-two controlled trials met the inclusion criteria, of which nineteen were randomised and three were non-randomised.

### 3.2. Characteristics of Included Studies

The characteristics of the studies included in this review are presented in Table 1. Most of the papers identified were published after 2010 (except Karanikas et al. [26] and Xiang et al. [27]), and seven of them were published in the last 3 years [28,29,30,31,32,33,34]. Fourteen studies were conducted in Asia (seven in China [27,29,32,34,35,36,37], six in Iran [28,30,31,33,38,39], and one in India [40]), seven in Europe (three in Italy [41,42,43], one each in Austria [26], Germany [44], Poland [45], and Romania [46]), and one in South America (Brazil [47]). The sizes of groups in the studies varied (15–183 subjects), but usually the number in the intervention groups did not exceed 50 people (except in three studies [32,35,37]), and the smallest intervention group consisted of 15 people [42]. The majority of the studies were conducted only among women [26,27,31,35,37,39,41,43,44,45,46]. In the studies included in the systematic review, the following DS were used: three studies examined the influence of vitamin D [31,38,39], twelve studies of selenium [26,29,32,34,35,36,40,41,44,45,46,47], and seven studies used other DS including zinc, magnesium, and vitamin A [30], Wobenzym vital [42], *Nigella sativa* [28], fermented papaya-based nutraceutical [43], genistein [37], synbiotic [33], and alpha-lipoic acid [27]. The studies included also differed in terms of the biomarkers tested—in addition to thyroid parameters, the authors analysed various inflammatory, oxidative stress, and/or antioxidant status parameters. 

### 3.3. Study Quality

Details of the quality assessment of the randomised studies are shown in Table S2. Most of the randomised controlled trials were considered to have major limitations (*n* = 13); only six studies [27,28,30,36,41,45] were assessed as those with minor limitations. Based on the CASP tool [23], the main identified problems in studies with major limitations were: not comprehensively reported effects of an intervention; no harms and costs were indicated in relation to intervention benefits; and not all studied groups had received the same level of care.

The results of the evaluation of non-randomised studies are shown in Table S3. Based on the ROBINS-I tool [25], in all non-randomised studies (*n* = 3), the main issue was bias in the measurement of outcomes, which could have affected the results evaluated. In two non-randomised studies, the design was open label [42,43], and only in one study did the participants not know whether they had received DS or placebo [40]. There was no bias in participant selection, deviations from the interventions intended, or the selection of reported results. Due to missing data in one study, estimation of the risk of bias was impossible, as the information available was insufficient for assessment. The overall risk of bias was considered moderate for all three studies.

### 3.4. Results of Vitamin D Supplementation

Only three randomised control trials examined the effect of vitamin D supplementation on the inflammation status of patients with AIT [31,38,39] (Table 2). All studies had major limitations.

#### 3.4.1. Vitamin D Status

In all studies, the duration of the intervention (3 months) and the doses of vitamin D3 (50,000 IU per week) were the same. The supplementation used increased 25-hydroxyvitamin D (25(OH)D) serum levels in all studies, but only in two studies [31,38] was the difference between the intervention and control groups statistically significant after the end of the intervention.

#### 3.4.2. Inflammation Parameters

In all three studies, none of the analysed parameters of inflammation differed between the intervention and control groups after the end of the intervention. Although, in the study by Robat-Jazi et al. [31], the levels of IFN-γ and TNF-α decreased statistically significantly in the intervention group after vitamin D supplementation, a similar association was found in the control group as well.

#### 3.4.3. Oxidative Stress and Antioxidant Status Parameters

There were no oxidative stress or antioxidant status parameters analysed in the studies.

#### 3.4.4. Thyroid Parameters

Of the three studies, only in one did the authors provide results for a single thyroid parameter, i.e., TSH, before and after the intervention [38]; in that study, the results did not indicate a statistically significant impact of vitamin D supplementation on TSH level in AIT patients.

### 3.5. Results of Selenium Supplementation

A summary of the results of 12 studies on selenium supplementation in relation to the parameters of inflammation, oxidative stress, antioxidant status, and the thyroid in an intervention and control group is presented in Table 3. The duration of the interventions varied from 3 to 12 months. The most used form of selenium was selenomethionine (five studies) [41,44,45,46,47] and selenium yeast tablets (5 studies) [29,32,34,35,36]. Most of the studies used a dose of 200 µg/day of selenium [26,29,34,35,36,40,45,47] or lower [32,41,44,46].

#### 3.5.1. Selenium Status

In eight studies, parameters related to nutritional selenium status were presented [26,29,34,35,36,44,46,47]; in six studies, a significant increase in selenium levels was observed after supplementation and in comparison to the control group at the end of the intervention [26,29,35,36,46,47]. In two studies, together with selenium concentration, the serum selenoprotein P (SePP) level was determined [29,44]. In contrast to the study by Pilli et al. [44], in the study by Hu et al. [29], the SePP level was statistically significantly higher in the intervention group compared to the control group after the supplementation.

#### 3.5.2. Inflammation Parameters

Six studies examined selenium supplementation in relation to its influence on inflammation parameters [26,32,36,41,44,45]. Krysiak and Okopień [45] have conducted a study with selenium alone and with selenium supplement and levothyroxine combined in AIT female patients. In comparison to the placebo group, significant differences were observed after selenium supplementation in terms of the high-sensitivity C-reactive protein (hsCRP), IL-2, IFN-γ, and TNF-α lymphocyte levels. Simultaneous use of selenium supplements and levothyroxine also had a statistically significant effect on IL-1β, IL-6, and monocyte chemotactic protein-1 (MCP-1). This study was one of three that explored the effects of selenium supplementation, with minor limitations.

In a study conducted by Pilli et al. [44] (with major limitations) with AIT patients, two doses of selenomethionine (80 and 160 μg/day) were administered. Both doses had a significant effect on serum levels of IFN-γ, TNF-α, and C-X-C motif chemokine ligand 11 (CXCL-11) 6 months after the introduction of supplementation, but after 12 months, the levels of these inflammatory markers increased and did not differ compared to the baseline. Serum CXCL-9 and CXCL-10 levels significantly differed between the intervention and the placebo group after the 12 month supplementation. Serum CXCL-9 significantly decreased by the 12th month with a dosage of 80 μg/day and by the 6th month with a dosage of 160 μg/day (without any further decrease in the 12th month), while serum CXCL-10 level (for both selenium dosages) decreased significantly after the end of the intervention (12th month, *p* < 0.05).

In a study by Sun et al. [32] in AIT patients, the serum levels of IL-2 and TNF-α significantly decreased after 3 months of selenium yeast supplementation, and the level of anti-inflammatory IL-10 significantly increased. These results were statistically significantly different from those obtained in the control group after the end of the intervention. Similarly, in a study by Yu et al. [36], also in AIT patients, it was observed that 3 month selenium yeast supplementation significantly decreased IL-2 levels but increased IL-10 levels. However, in comparison to the control group, only the IL-2 level showed a significant difference after the end of the intervention (*p* < 0.001). Of the two studies, only the study by Yu et al. [36] had minor limitations.

In the other three studies involving AIT patients, inflammation parameters did not differ between the intervention and control groups after the end of the intervention [26,34,41]. One of these studies had minor limitations [41].

#### 3.5.3. Oxidative Stress Parameters

Only three studies have examined the impact of selenium supplementation on oxidative stress parameters in patients with HT [40] or AIT [34,35] (Table 3). Two studies had major limitations [34,35], and one had a moderate risk of bias [40]. In all studies, a statistically significant decrease in serum malondialdehyde (MDA) levels was observed after the supplementation, but the results were not different compared to the placebo groups.

#### 3.5.4. Antioxidant Status Parameters

Among the antioxidant status parameters, the activity of glutathione peroxidase (GPx) was assessed in five studies with AIT patients [29,35,44,46,47] (all with major limitations), while superoxide dismutase (SOD) activity and total antioxidant capacity (TAC) were assessed in only one study [34]—see Table 3.

In contrast to the results obtained by Preda et al. [46], in the study by de Farias et al. [47], serum activity of GPx1 increased significantly after 3 months of selenium supplementation and was significantly different compared to the placebo group after this intervention. Additionally, Hu et al. [29] and Wang et al. [35] observed statistically significant increased GPx3 activity after 6 months of selenium yeast supplementation, but the difference between the intervention and the control group after the end of the intervention was significant only in the Hu et al. [29] study. In contrast, Pilli et al. [44] did not observe significant changes in GPx3 activity after supplementation with 80 μg selenomethionine (the authors did not determine GPx3 activity in the group with 160 μg selenium supplementation).

Results obtained by Tian et al. [34] indicate that 3-month selenium yeast supplementation statistically significantly increased the levels of TAC and SOD activity in the intervention group. Moreover, after the intervention, these parameters differed between the intervention and control groups; however, the authors did not present a *p*-value for such a comparison.

#### 3.5.5. Thyroid Parameters

The influence of selenium supplementation on the thyroid was assessed in all studies, but the thyroid parameters assessed varied (Table 3).

In seven studies [26,29,34,35,41,44,45], selenium supplementation did not affect TSH levels; in two studies [46,47], the intervention used increased TSH levels, while in the other two studies [32,40], it decreased them. Although a significant decrease in serum TSH levels was observed in the studies by Chakrabarti et al. [40] and Sun et al. [32], similar changes were also observed in the control groups. After the end of the intervention, no significant difference between the intervention and control groups was observed in the study by Sun et al. [32], while in the study by Chakrabarti et al. [40], this information was not available.

Seven studies [29,32,34,35,36,45,46] demonstrated a significant decrease in TPO-Ab titer after selenium supplementation in the intervention groups, with the largest decrease observed in the study by Hu et al. [29]. However, only three studies [32,36,45] showed a significant difference in TPO-Ab titer between the intervention and control groups after the end of the intervention, and two of them had minor limitations [36,45].

### 3.6. Results of the Studies with Other DS

Table 4 presents the outcomes of studies on DS supplementation with ingredients other than vitamin D and selenium. Two studies used multicomponent DS [30,42], making it difficult to determine which specific ingredient had a potential effect. The study by Nordio and Basciani [42] had a moderate risk of bias, while the study by Rabbani et al. [30] had only a minor limitation. The study by Farhangi and Tajmiri [28] with *Nigella sativa* powder and the study by Xiang et al. [27] (alpha-lipoic acid) had minor limitations, while the studies by Zhang et al. [37] (genistein) and Talebi et al. [33] (synbiotic) had major limitations. The study by Tomella et al. [43] (fermented papaya-based nutraceutical) had a moderate risk of bias.

#### 3.6.1. Inflammation, Oxidative Stress, and Antioxidant Status Parameters

The study with minor limitations by Rabbani et al. [30] examined a combination of vitamin A, zinc, and magnesium on hsCRP, MDA, and TAC in HT patients. It was found that hsCRP levels decreased after supplementation in the treatment group, and the changes in this parameter compared to the control group were statistically significantly different after the end of the intervention. There was also a significant difference in TAC level between the intervention and control groups after the end of the intervention; in the intervention group, TAC did not change, while in the control group, it decreased.

In another study, supplementation with Wobenzym alone and in combination with levothyroxine in AIT patients [42] decreased CRP levels. However, the authors did not compare the levels of these parameters with the control group after the end of the intervention.

In the study conducted by Farhangi and Tajmiri [28], 8 week administration of powdered *Nigella sativa* seeds in AIT patients resulted in decreasing serum MDA levels and increasing TAC levels and SOD activity but not GPx activities. However, after the end of the intervention, the parameters did not differ in comparison to the placebo group.

Zhang et al. [37] observed a significant decrease in serum IL-2 level (but not in IL-4, IL-6, or TNF-α levels) and an increase in IFN-γ level after one month of genistein supplementation in AIT patients. In contrast, in another study, 8 week symbiotic supplementation increased hsCRP levels in HT patients, but after the end of the supplementation, the results in the intervention vs. control group did not differ [33].

Other studies did not observe a significant effect of fermented papaya-based nutraceutical supplementation [43] and alpha-lipoic acid supplementation [27] on parameters related to oxidative stress and antioxidant status in HT [43] or AIT [27] patients.

#### 3.6.2. Thyroid Parameters

A significant decrease in TSH levels was observed after supplementation with Wobenzym vital, genistein, *Nigella sativa*, and synbiotic [28,33,37,42], but in the study by Nordio and Basciani [42], this difference was only observed in the group supplemented with Wobenzym vital and L-thyroxine (LT4). Serum fT3 or T3 levels increased significantly after *Nigella sativa* [28] and synbiotic [33] supplementation. Serum fT4 levels demonstrated a significant increase following supplementation with a combination of vitamin A, zinc, and magnesium [30], as well as genistein [37]. A significant decrease in TPO-Ab titer was observed after supplementation with *Nigella sativa* [28] and genistein [37]. In the study with Wobenzym [42], supplementation in combination with levothyroxine significantly decreased only high-sensitivity human thyroglobulin antibodies (HTg-Ab) titers while not impacting fT3 and TPO-Ab. After the intervention, the parameters were lower in the intervention group compared to the control group; however, the authors did not present *p*-values for such a comparison.

## 4. Discussion

To summarise the results of the studies included in the systematic review: (1) based on a limited number of studies, it was found that there is a lack of influence of vitamin D supplementation on inflammatory parameters; none of the studies analysed oxidative stress and antioxidant status parameters, and only one provided results for a single thyroid parameter (i.e., TSH) after the intervention; (2) some evidence was found that selenium supplementation may decrease inflammation and improve thyroid parameters; only a few studies with inconsistent results assessed oxidative stress based on one parameter (i.e., MDA) and antioxidant status based on a few parameters; and (3) other supplements (such as zinc + magnesium + vitamin A, Wobenzym vital, *Nigella sativa* powder, or genistein) may potentially reduce inflammation and oxidative stress and improve thyroid parameters as well as increase antioxidant status, but the quality of these studies was low and each of the supplements was examined in only one study.

Hypothetically, the use of DS may be beneficial in AIT and/or HT patients with nutritional deficiencies. These diseases are associated with various nutritional deficiencies [20]. For example, vitamin D deficiency was found in 96.1% of AIT patients [48]; the prevalence of subclinical or overt HT and AIT was highest in the bottom quintile of serum selenium (<47 µg/L) [49]; a lower zinc serum level (<80 µg/dL) was observed in 40% of patients with overt HT and 30% of patients with subclinical HT [50].

In three randomised control trials, vitamin D supplementation did not have an impact on inflammatory and thyroid parameters. Those results can be linked to the small group sizes in all studies and the short duration of the interventions. Furthermore, all three studies were assessed as having major limitations. In two of them, the after-intervention values of the parameters as well as the *p*-values for their changes were not provided; thus, it was not possible to establish the changes.

Similar conclusions were reached by Jiang et al. [51] in a meta-analysis of RCTs in patients with AIT. The authors concluded that although vitamin D supplementation has not been associated with improvement of thyroid functions in patients with AIT [51], at the cellular level, it has an anti-inflammatory effect by reducing proinflammatory cytokine production from macrophages and T cells. Sufficient vitamin D status could regulate T cell proliferation, while insufficiency paired with autoimmune diseases is associated with increased B cell proliferation and autoantibody production [52,53]. This could explain the reason why a decrease in the level of TPO-Ab titer was observed in Nodehi et al. [39] and Robat-Jazi et al. [31], even though it is not linked to changes in other thyroid parameters. There are multiple possible reasons explaining vitamin D’s role in AIT, and one of them is that the secretion of large amounts of immunoglobulin G (IgG), E (IgE), and other immunoglobulins triggered by vitamin D insufficiency causes damage to thyroid cells. The expression of the vitamin D receptor (VDR) in naïve and memory T cells indicates that vitamin D can directly impact T cells and regulate their responses [54]. There is evidence that vitamin D, through the inhibition of the clusters of differentiation 4 positive (CD4+) T cells to Th1 cell conversion, is able to inhibit the production of IFN-γ [55]. A similar mechanism can be observed regarding the impact of vitamin D on the transformation of CD4+ T cells into Th17 cells, which have pro-inflammatory properties and secrete TNF-α [54]. This mechanism provides an explanation for the findings of the study conducted by Robat-Jazi et al. [31], who observed a significant difference in IFN-γ and TNF-α levels.

Of the twelve studies in which selenium supplements were used, and in those where *p*-values were available, the difference between the intervention and control groups in inflammatory biomarkers was statistically significant in six of them [29,32,36,44,45,47], and it concerned a total of nine different parameters (hsCRP, IL-2, IL-10, IFN-γ, TNF-α, GPx1, GPx3, CXCL-9, and CXCL-10). However, among those six studies, only two were considered to have minor limitations [36,45].

Among inflammatory markers, the IL-2 level decreased significantly after the intervention in three studies [32,36,45]. Yu et al. [36] stated that the reason behind it is that selenium has a regulatory influence on cytokine production. Krysiak and Okopień [45] also suggested this, which, at the same time, is a possible explanation for the decrease in TPO-Ab titers. Another cytokine that had decreased significantly in two studies was TNF-α [32,45]. In the study by Krysiak and Okopień [45], this change was observed only in lymphocyte release, while in the study by Sun et al. [32], it was not specified. Both parameters belong to Th1 cytokines and have pro-inflammatory properties. Furthermore, in Sun et al., the serum IL-10 level significantly increased after the end of the intervention, which is also beneficial due to the anti-inflammatory properties of IL-10 and Th2 cytokines. Considering the potential shift in Th1/Th2 balance, it can explain the decrease in TPO-Ab titers in Sun et al. [32], because the imbalance of those cytokines is associated with AIT [56]. Among the three studies [26,44,45] that assessed IFN-γ, only in the study by Krysiak and Okopień [45] was the difference between groups after the end of the intervention significant. A possible explanation was suggested by Krysiak and Okopień [45], who compared their study to that of Karanikas et al. [26] and pointed out that in their study, baseline disease activity was higher and individual baseline selenium levels also differed. Moreover, in the study by Krysiak and Okopień [45], the number of participants in the trial was higher than in the other two, and this probably also had an impact on the achievement of significant results. Furthermore, in the study by Pilli et al. [44], the levels of two inflammatory parameters returned to their baseline values during the intervention, but the duration of this intervention was longer than in the studies by Krysiak and Okopień [45] or Sun et al. [32], which may also have influenced the results. Pilli et al. [44] also observed a significant decrease in CXCL-9 and -10 levels, but not in CXCL-11. This may indicate that while selenomethionine has an immunomodulatory effect, it is also selective in its action, which was previously noted by Krysiak and Okopień [45]. This beneficial effect of selenium supplementation might be linked with the role of selenoenzymes in immunoregulatory processes, which involve T-cell activity and the production of cytokines [57].

In the study by de Farias et al. [47], a significant difference between the intervention and control groups after the intervention was found for serum GPx1. Similarly, in the study conducted by Hu et al. [29], the GPx3 activity exhibited a significant difference. The change in these parameters is due to their specific character. They belong to glutathione peroxidases, which contain selenium, making them selenoproteins. The role of GPx1 is to protect the intracellular compartment from excess hydrogen peroxide, thereby protecting thyrocytes. Likewise, the thyroid gland is protected from hydrogen peroxide by GPx3 [58,59]. According to De Farias et al. [47], TPO-Ab titer may decrease, and this is linked to selenium level improvement and reduction of damages caused by reactive oxygen species as an effect of an increase in GPx1 activity. This can possibly also explain the results of the study by Hu et al. [29], in which the difference between the reduction of TPO-Ab titers in the intervention and control groups after the end of the intervention was statistically significant.

Three studies investigating the relation between inflammation status and thyroid parameters [29,32,36] used selenium yeast tablets, while two studies [45,47] used selenomethionine. It is suggested that selenomethionine might be a more effective form of supplement than non-organic forms [60]. Considering that selenium yeast contains up to 90% of selenium in the selenomethionine form [61], in this systematic review, significant results were noted for those studies where selenomethionine was used. However, it should not be overlooked that in the other five studies [34,35,41,44,46] that used selenium yeast or selenomethionine, the results were not that promising. Similarly, in the case of the results of a meta-analysis of eleven studies [62], it is not possible to unequivocally conclude whether selenium supplementation should be a routine recommendation for AIT patients based on the findings of this review. Selenium supplementation should be considered individually based on the body’s selenium status. Among members of the European Thyroid Society, 65% recommend selenium supplements to their patients with AIT, although only 20% claimed that the available evidence validates the use of this ingredient [63].

Of the seven [27,28,30,33,37,42,43] studies with other DS, only two (fermented papaya-based nutraceutical, combination of vitamin A, zinc, and magnesium) [30,43] observed significant post-intervention differences between the intervention and control groups based on available data. Moreover, only two of them were assessed as having minor limitations [28,30], which may have been reflected in the relevance of the results. Other studies had major limitations [33,37] or a moderate risk of bias [42,43].

In the study by Rabbani et al. [30], the level of hsCRP decreased significantly, fT4 increased significantly, while oxidative stress (MDA) and antioxidant status (TAC) parameters did not change after combining vitamin A, zinc, and magnesium supplementation in HT patients. It is not possible to say conclusively what mechanism of action caused this effect because the supplement used was multicomponent and the interactions between its components are difficult to explain.

*Nigella sativa* powder had a significant impact on oxidative stress and antioxidant status parameters within the intervention group, but there was no difference between the intervention and control groups after the end of the intervention [28]. The potential antioxidant properties of this supplement have been linked to its ingredients, specifically dithymoquinone and thymol. Consequently, changes in thyroid parameters may be observed.

In the study by Nordio and Basciani [42], Wobenzym vital had a similar effect on inflammatory parameters by itself and in combination with LT4. However, a significant influence on serum TSH level or TPO-Ab titer was only observed in the group that was administered Wobenzym vital and LT4, which makes the impact of this DS unclear.

The observed increase in serum CRP level after synbiotic supplementation can be attributed to the high concentration of *Lactobacillus Casei* in this DS. However, altering the composition may yield different results, highlighting the need for further studies [33]. Those results are consistent with the meta-analysis by Kazemi et al. [64], where they indicated that *L. Casei* could increase CRP levels. Based on the data collected, it is not possible to make a recommendation to use these food supplements for improving inflammatory parameters, oxidative stress, or antioxidant status.

### Strengths and Limitations

To the best of our knowledge, this is the first systematic review to compile studies concerning such a comprehensive range of dietary supplements. In addition to the use of selenium and vitamin D supplements, it also considers the use of other supplements not typically associated with HT or AIT. The strength of this review is compliance with PRISMA guidelines for reporting systematic reviews [22], as well as database searches and quality assessments of the studies by two independent researchers. The inclusion criteria were clearly defined, and the researchers followed them strictly. The included studies were conducted within different populations; country was not an exclusion criterion, and there was no minimum number of biomarkers that had to be assessed in a study. Thanks to this, it was possible to provide a complete picture and compare the efficacy of DS in reducing inflammation, oxidative stress, and improving antioxidant status in relation to their potential effects on thyroid parameters.

We are aware of several limitations in this paper as well as the studies included in this review. A relatively small number of studies were included in the review, and most of them had major limitations or a moderate risk of bias. The included studies differed in terms of analysed parameters; data were often presented in different ways; some studies lacked specific results (like mean or median) and provided incomplete statistical analyses (e.g., *p*-values were not presented). These often make it difficult or impossible to directly compare the results and conduct a meta-analysis. In the included studies, the group sizes were relatively small, and the interventions usually lasted no longer than 6 months. The studies differed in terms of LT4 treatment; in some of them, the inclusion criterion was no LT4 treatment before the start of the trial [29,32,34,40,41,42,45,46], while in nine studies, treatment before the start of the trial was obligatory [26,30,31,33,36,38,39,44,47]. This may also have influenced the results of these studies and, consequently, the conclusions of this systematic review.

## 5. Conclusions

Based on the results of the systematic review conducted, no recommendations can be put forward regarding the use of DS in HT and/or AIT patients. The limited evidence suggested that selenium supplementation might be beneficial considering its influence on reducing inflammation status and improving thyroid parameters; however, such an intervention should be launched based on selenium deficiency. Due to the lack of studies on the efficiency of vitamin D supplementation in HT/AIT patients in terms of antioxidant status and oxidative stress, as well as the lack of influence on inflammatory parameters in limited studies, conclusions cannot be drawn. However, consideration should be given to improving vitamin D status in deficient populations, regardless of potential thyroid gland improvement [65].

These inconclusive results of the systematic review highlight the need for further long-term, high-quality randomised controlled trials, particularly due to common nutritional deficiencies in patients with HT or AIT. The use of DS should not be recommended without a prior assessment of nutritional status or dietary habits. The safe use of dietary supplements should aim at correcting nutritional deficiencies and improving parameters related to inflammation, oxidative stress, or antioxidant status in relation to improving thyroid gland function in hypothyroidism or Hashimoto’s thyroiditis.

## Figures and Tables

**Table 1:** Characteristics of the studies included in the systematic review.

Authors, Publication Year	Intervention/Control Group Size (Country)	Inclusion Criteria	Exclusion Criteria	Age Intervention /Control Group (Years; Mean ± SD)	Inflammatory Parameters	Oxidative Stress Parameters	Antioxidant/Nutritional Status Parameters	Thyroid Parameters
Vitamin D								
Anaraki et al., 2017 [38] Randomised, double-blind, placebo-controlled clinical trial	30/26 (Iran)	Female/Male, AIT with/without HT Intervention (vitamin D): adults; hypothyroidism or euthyroidism with positive TPO-Ab; stable on LT4 at least for 6 months/or a mild hypothyroidism on enrolment TSH < 15 mU/L Control (placebo): the same criteria as in the intervention group	Renal or liver disease; cancer; pregnancy; severe weight loss; immunosuppressive medication, insulin, sulfonamides; any supplements.	43.55 ± 1.56 /44.12 ± 1.56	CRP	—	25(OH)D	TSH
Nodehi et al., 2019 [39] Randomised, double-blind, placebo-controlled trial	17/17 (Iran)	Female, AIT Intervention (vitamin D): Hashimoto’s thyroiditis, age 20–45 years; constant doses of LT4. Control (placebo): the same criteria as in the intervention group	History of other specific diseases; receiving vitamin supplements in the last 3 months.	36.4 ± 5.2 /35.9 ± 7.8	IL-10, IFN-γ, IL-17, IL-4	—	25(OH)D	TSH, TPO-Ab, Tg-Ab
Robat-Jazi et al., 2022 [31] Randomised, double-blind, placebo-controlled trial	18/20 (Iran)	Female, AIT Intervention (vitamin D): Hashimoto’s thyroiditis, age 18–48 years; BMI 18.5–30; treatment with LT4 for 6 months Control (placebo): the same criteria as in the intervention group	Severe hepatic, biliary, pancreatic, and fatty liver disease; diseases affecting the balance of CD4+ T cells (asthma, active viral diseases); autoimmune diseases; malnutrition; obesity; treatment with vitamin D supplementation within 3 months prior; pregnancy, lactation; alcoholism; history of stroke or MI.	36.4 ± 5.2 /35.9 ± 7.8	IFN-γ, TNF-α	—	25(OH)D	TSH, TPO-Ab, Tg-Ab
Selenium								
Chakrabarti et al., 2016 [40] Placebo-controlled trial	30/30 (India)	Female/Male, HT Intervention (selenium): adults; overt hypothyroidism, based on the low fT4 level with elevated TSH level; treatment-naïve Control (placebo): the same criteria as in the intervention group	Patients with hypothyroidism but on treatment with LT4; taking lipid-lowering drugs and antioxidant supplements; smokers and alcoholics; pregnancy; patients with hypertension, diabetes mellitus, hepatic or renal impairment, CAD, or heart failure.	34.63 ± 10.94 /39.57 ± 13.62	—	MDA	—	TSH, fT4
de Farias et al., 2015 [47] Prospective, randomised, double-blind, placebo-controlled trial	23/30 (Brazil)	Female/Male, AIT Intervention (selenium): Hashimoto’s thyroiditis is established by an increased level of TPO-Ab (>100 UI/mL), a normal or increased Tg-Ab level (>100 UI/mL), thyroid hypoechogenicity on high-resolution ultrasound, a normal or elevated TSH level, and normal fT4 Control (placebo): the same criteria as in the intervention group	Use of immunosuppressive or anti-inflammatory drugs, supplements containing micronutrients, antidepressants, anticonvulsants, or antiarrhytmic drugs; health conditions that may interfere with gastrointestinal absorption or with the metabolism of selenium; and diabetes.	48 (20–58) /44 (21–56) *	—	—	GPx1, Se	TSH, TPO-Ab, Tg-Ab
Esposito et al., 2017 [41] Prospective, randomised, blinded, placebo-controlled trial	38/38 (Italy)	Female, AIT Intervention (selenium): newly diagnosed subjects with elevated plasma TPO-Ab and Tg-Ab above 350 IU/ml; thyroid parenchyma heterogeneity with reduced echogenicity; normal TSH, fT3, and fT4 serum levels; without LT4 treatment Control (placebo): the same criteria as in the intervention group	Male; living outside the Campania district area; hyperthyroidism with antithyroid drugs; hypothyroidism with LT4 treatment; other medication that can influence thyroid and immunity status; pregnancy.	40.0 ± 12.1 /46.0 ± 14.1	CXCL-10	—	—	TSH, fT3, fT4, TPO-Ab, Tg-Ab
Hu et al., 2021 [29] Randomised, controlled study	43/47 (China)	Female/Male, AIT Intervention (selenium): age 18–65 years; positive serum TPO-Ab (>34 IU/mL) or/and Tg-Ab (>115 IU/mL); diffuse thyroid disease and heterogeneous echogenicity on ultrasonography; euthyroid or subclinical hypothyroid; newly diagnosed HT patients not receiving LT4 replacement, immunomodulator, vitamins, and other trace elements Control (none): the same criteria as in the intervention group	Previous treatments with immune suppressors or modulators; combined with other autoimmune diseases; pregnancy or lactation in women, or planning pregnancy within 6 months; unability to take medication on time; participation in another clinical trial, informed consent withdrawn	39.4 ± 12.0 /37.8 ± 11.2	—	—	GPx3, Se, SePP	TSH, fT3, fT4, TPO-Ab, Tg-Ab
Karanikas et al., 2008 [26] Randomised, placebo-controlled trial	18/18 (Austria)	Female, AIT Intervention (selenium): verified Hashimoto’s thyroiditis; LT4 substitution; positivity for TPO-AB, negativity for anti-thyrotropin (TSH) receptor antibodies; thyroid ultrasound imaging suggestive for a chronic thyroiditis (typical hypoechogenicity); no clinical history of hyperthyroidism; no treatment with drugs known to induce thyroid dysfunction (cytokines, lithium, amiodarone); no pregnancy in the last 12 months prior to enrolment Control (placebo): the same criteria as in the intervention group	Not meeting inclusion criteria	Mean for all: 47	IL-2, IL-4, IL-10, IL-13, IFN-γ, TNF-α	—	Se	TSH, fT4, TPO-Ab
Krysiak and Okopień, 2011 [45] Randomised, double-blind, placebo-controlled trial	Two intervention groups: SE: 42; LT4 + Se: 42 Control with LT4:41 Placebo:40 (Poland)	Female, AIT Intervention (selenium): age 18–60 years; newly diagnosed and previously untreated Hashimoto’s thyroiditis; positive TPO-Ab level (>100 U/mL); reduced echogenicity of the thyroid parenchyma on ultrasonography; TSH level < 4.0 mU/L; normal level of free T4 and free T3 Control (placebo, LT4): the same criteria as in the intervention group	Any acute/chronic inflammatory processes; other autoimmune disorders; positive serum antibodies against the TSH receptor; current treatment with thyroid hormones; treatment with drugs that may affect inflammatory processes in the vascular wall; treatment with drugs known to affect thyroid hormones or to interact with LT4 and selenomethionine; BMI > 40 kg/m^2^; Turner or Down syndrome; severe cardiovascular disease, diabetes, impaired glucose tolerance or impaired fasting glucose; impaired renal or hepatic function; pregnancy or lactation; poor patient compliance	40 ± 4 /37 ± 3 /39 ± 4 /38 ± 3	IL-1β, IL-2, IL-6, IFN-γ, TNF-α, hsCRP MCP-1/CCL-2	—	—	TSH, fT3, fT4, TPO-Ab, Tg-Ab
Pilli et al., 2015 [44] Randomised, placebo-controlled trial	Se (80 μg/day): 20 Se (160 μg/day): 20 / 20 (Germany)	Female, AIT Intervention (selenium): Hashimoto’s thyroiditis (present of elevated TPO-Ab or/and Tg-Ab serum levels (≥100 U/ml), characteristic thyroid ultrasound pattern (scattered or widespread hypoechogenicity), normal thyroid function; no previous treatment with LT4 replacement therapy Control (placebo): the same criteria as in the intervention group	Not meeting inclusion criteria	48.8 ± 14 46.9 ± 7.6 /43.0 ± 11.2	IFN-γ, TNF-α, CXCL-9, CXCL-10, CXCL-11	—	GPx3, Se, SePP	TSH, fT3, fT4, TPO-Ab, Tg-Ab, Thyroid volume, Thyroid echogenicity
Preda et al., 2017 [46] Randomised, placebo-controlled trial	50/50 (Romania)	Female, AIT Intervention (selenium): adults; euthyroid Hashimoto’s thyroiditis, detectable TPO-Ab levels (>35 UI/mL), normal TSH level (0.4–4 uIU/mL) Control (placebo): the same criteria as in the intervention group	Not meeting inclusion criteria	46.2 ± 12.5 /50.5 ± 13.5	—	—	GPx1, Se	TSH, TPO-Ab
Sun et al., 2021 [32] Randomised-controlled trial	69/69 (China))	Female/Male, AIT with HT Intervention (selenium): age 20–64 years, no serious cardiovascular, cerebrovascular, hepatic, renal, and hematopoietic system disease; psychiatric disorders; not receiving LT4; immunomodulatory preparations and selenium preparations in the 1 month prior to enrolment; no comorbid autoimmune disease Control (none): the same criteria as in the intervention group	Obvious gastrointestinal disorders that cause problems with the use of oral medication; pregnancy or lactation; planning to become pregnant within 6 months; allergies or hypersensitivity to the medication used in the study; other combined thyroid disorders	42.6 ± 5.3 /41.6 ± 6.1	IL-2, IL-10, TNF-α	—	—	TSH, TT3, TT4, TPO-Ab, Tg-Ab
Tian et al., 2020 [34] Randomised, placebo-controlled trial	16/16 (China)	Female/Male, AIT Intervention (selenium): euthyroid, newly diagnosed with Hashimoto’s thyroiditis, age ≥ 18 years old Control (placebo): the same criteria as in the intervention group	Nonthyroidal disorders, including cancer, hypertension, diabetes mellitus, coronary artery disease, chronic kidney disease, liver diseases, heart failure, cerebrovascular disease, rheumatism, and smokers; use of other antioxidant agents or vitamin supplements within the past 6 months; pregnancy	42.3 ± 5.4 /41.6 ± 6.8	—	MDA	TAC, SOD, Se	TSH, TPO-Ab, Tg-Ab
Wang et al., 2018 [35] Randomised, double-blind, placebo-controlled trial	181 (including 153 subclinical and 28 clinical AIT) 183 placebo (including 160 subclinical and 23 clinical AIT) (China)	Female, AIT Intervention (selenium): age 15–70 years; elevated serum TPO-Ab (>300 IU/mL); thyroid hormone levels within the reference range—TSH may be above the upper normal level (subclinical hypothyroidism); without any medication that can affect either Se bioavailability or peripheral conversion of T4 to T3 or medications that could influence thyroid autoimmunity; nonsmokers Control (placebo): the same criteria as in the intervention group	Not meeting inclusion criteria	40.3 ± 12.2 /43.1 ± 11.6	—	MDA	GPx3, Se	TSH, fT4, TPO-Ab
Yu et al., 2017 [36] Open-label, randomised controlled trial	34/36 (China)	Female/Male, AIT Intervention (selenium): Hashimoto’s thyroiditis Control (none): the same criteria as in the intervention group	Residence in an iodine deficiency area with goitre; treatment with immune suppressors or modulators, selenium or other antioxidants, within one month; other autoimmune diseases; severe liver, kidney, GI tract, blood system, brain, circulation system, or blood-vessel system illness; pregnancy or lactation or planning pregnancy within 6 months; mental or nervous system disease not allowing to cooperate or take medication on time, and abuse of drugs or other substances; malignant tumour; allergy to the test drug; surgery or other stressful conditions; participation in another clinical trial.	34.12 ± 12.7 /39.50 ± 15.1	IL-2, IL-10	—	Se	TPO-Ab, Tg-Ab
Other supplements								
Rabbani et al., 2021 [30] Randomised, double-blind, placebo-controlled trial	44/44 (Iran)	Female/Male, HT Intervention (zinc, magnesium, vitamin A): hypothyroidism; age 20–65 years; BMI ≤ 35 kg/m^2^; no serious medical illness (e.g., diabetes); no uncontrolled hypertension or gastrointestinal diseases. Control (placebo): the same criteria as in the intervention group	Pregnancy or lactation; smoking; drinking alcohol; consumption of anti-inflammatory medication; use of dietary supplements containing Zn, Mg, and vitamin A during the past 3 months; unwillingness to continue the study; and taking less than 80% of supplements in any follow-up visit	42.47 ± 10.7 /48.33 ± 11.0	hsCRP	MDA	TAC	TSH, fT3, fT4, TT4
Nordio and Basciani, 2015 [42] Non-randomised placebo-controlled trial	Wob:15 LT4 + Wob:15 /15 (Italy)	Female/male, AIT Intervention (Wobenzym): age 18–80 years; Hashimoto’s thyroiditis, with or without hypothyroidism; no serious illnesses. Control (placebo): the same criteria as in the intervention group	Use of cortisone, NSAIDs, antiinflammatory agents, and anticoagulants; smokers; alcohol or drug use; pregnancy or lactation	All participants: 44.3 ± 6.8	CRP	—	—	TSH, fT3, TPO-Ab, HTg-Ab, HTG
Farhangi and Tajmiri, 2020 [28] Double-blinded, placebo-controlled randomised trial	20/20 (Iran)	Female/Male, AIT Intervention (*Nigella sativa*): age 20–50 years, diagnosed with Hashimoto’s thyroiditis. Control (placebo): the same criteria as in the intervention group	Taking any nutritional supplements for at least 3 months prior to the study; any history of autoimmune disease, cardiovascular events, or other thyroid abnormalities; dietary regimens during and 3 months before recruitment into the trial	35.70 ± 8.2 /33.95 ± 8.7	—	MDA	TAC, SOD, GPx	TSH, T3, T4, TPO-Ab,
Zhang et al., 2017 [37] Randomised, double-blind placebo-controlled clinical trial	102/116 (China)	Female, AIT Intervention (genistein): Hashimoto’s thyroiditis patients; age 20–60 years; normal fT3, fT4 levels, with or without LT4 therapy; normal or elevated but <20 mU/L TSH level; elevated serum TPO-Ab (>100 U/mL) Control (placebo): the same criteria as in the intervention group	Treatment with immunoregulators; acute infections or other chronic inflammatory diseases; thyroid nodules; thyroid hypoplasia; prior treatment with radioiodine; pregnancy; serious illness (such as cancer, kidney, or liver failure)	42.9 ± 10.2 /41.4 ± 9.3	IL-2, IL-4, IL-6, IL-10, IFN-γ, TNF-α	—	—	—
Tomella et al., 2014 [43] Placebo-controlled trial	30/39 (Italy)	Female, HT Intervention (fermented papaya-based nutraceutical): patients treated for subclinical or mild hypothyroidism; age 18–55 years; not on a birth control pill; not taking a soy supplement. Control (placebo): the same criteria as in the intervention group	Main chronic diseases, relevant medications, major dyslipidemia disorders, heavy physical activity, and psychiatric disorders	N/A	—	MDA	L-HPX, SOD, GPx	—
Talebi et al., 2020 [33] Randomised, double-blind, placebo-controlled	30/30 (Iran)	Female/Male, HT Intervention (synbiotic): age 18–65 years, primary hypothyroid patients with more than one year of levothyroxine therapy, at least one year of thyroid-stimulating hormone (TSH) levels in the normal range with a stable dose of levothyroxine; no thyroidectomy for thyroid cancer treatment; not having acute or chronic infectious diseases; not taking drugs that effect the metabolism or absorption of levothyroxine; not using antibiotics for at least 3 months before intervention; no history of smoking, alcohol, or drug abuse; no pregnancy or lactation Control (placebo): the same criteria as in the intervention group	Not meeting inclusion criteria	42.37 ± 2.2 /43.96 ± 1.8	CRP	—	—	TSH, fT3, TPO-Ab
Xiang et al., 2010 [27] Randomised controlled trial	20/18 (China)	Female, AIT Intervention (alpha-lipoic acid): age 46–68 years; newly diagnosed Hashimoto’s thyroiditis (elevated TSH levels (> 5.5 mU/L, normal fT3 and fT4 values), positive for TPO-Ab and Tg-AB. Control (none): the same criteria as in the intervention group	Obesity, smoking, thyroid operation, artery disease, other diseases, taking drugs like oestrogen supplements, thyroxine, diuretics, antihypertensive, hypolipidemic, etc.	57 ± 9 /56 ± 8	CRP	—	—	TSH, fT3, fT4, TPO-Ab, Tg-Ab

* Median (range); HT = hypothyroidism; AIT = autoimmune thyroiditis; TSH = thyroid-stimulating hormone; fT3 = free triiodothyronine; T3 = triiodothyronine; TT3 = total triiodothyronine; fT4 = free thyroxine; T4 = thyroxine; TT4 = total thyroxine; TPO-Ab = thyroid peroxidase antibodies; Tg-Ab = thyroglobulin antibodies; HTG = High-sensitivity human thyroglobulin; HTg-Ab = High-sensitivity human thyroglobulin antibodies; LT4 = L-thyroxine; 25(OH)D = 25-hydroxyvitamin D; CRP = C-reactive protein; hsCRP = high-sensitivity C-reactive protein; IL-1β = interleukin-1β; IL-2 = interleukin-2; IL-4 = interleukin-4; IL-6: interleukin 6; IL-10 = interleukin-10; IL-13 = interleukin-13; IL-17 = interleukin 17; IFN-γ = interferon gamma; TNF-α = tumour necrosis factor α; MCP-1/CCL-2 = monocyte chemotactic protein-1/chemokine (C-C motif) ligand 2; CXCL-9 = C-X-C motif chemokine ligand 9; CXCL-10 = C-X-C motif chemokine ligand 10; CXCL-11 = C-X-C motif chemokine ligand 11; MDA = malondialdehyde; TAC = total antioxidant capacity; SOD = superoxide dismutase GPx1 = glutathione peroxidase 1; GPx3 = glutathione peroxidase 3; MDA = malondialdehyde; GPx = glutathione peroxidase; TAC = total antioxidant capacity; SOD = superoxide dismutase; L-HPX = plasma hemopexin; Se = selenium; SePP = selenoprotein P; Wob = Wobenzym (mixture of plant-based enzymes, bioflavonoids, vitamin C, D, and E); N/A = not detailed results available (information provided in the text of the paper).

**Table 2:** Vitamin D concentration (25(OH)D) vs. inflammatory status and thyroid parameters in an intervention and control group before and after vitamin D supplementation.

Authors	Vitamin D (Cholecalciferol) Dose (IU/Week) (Duration of Intervention)	Parameter (Unit)	Intervention Group Baseline vs. after Supplementation (Mean ± SD)	*p*-Value	Control Group Baseline vs. after Placebo (Mean ± SD)	*p*-Value	*p*-Value after Supplementation vs. Control
Anaraki et al. [38]	50,000 (3 months)	25(OH)D (ng/mL)	12.8 ± 0.7 vs. 45.5 ± 1.8	0.001	13.3 ± 0.9 vs. 14.9 ± 1.1	0.090	0.001
		CRP (mg/dL)	1.1 ± 0.1 vs. 1.1 ± 0.1	0.890	1.2 ± 0.1 vs. 1.2 ± 0.1	0.970	0.790
		TSH (mU/L)	3.3 ± 0.5 vs. 3.9 ± 0.8	0.405	3.4 ± 0.4 vs. 2.7 ± 0.4	0.092	0.160
Nodehi et al. [39]	50,000 (3 months)	25(OH)D (ng/mL)	26.0 ± 14.6 vs. 42.3 ± 16.0 *	0.002	29.8 ± 12.3 vs. 36.2 ± 15.2 *	NS	0.230
		IL-4 (MFI)	22.2 ± 3.7 vs. 20.2 ± 0.9 *	N/A	37.5 ± 9.6 vs. 19.8 ± 0.8 *	N/A	0.601
		IL-10 (MFI)	144 ± 14.7 vs. 183 ± 18.0 *	N/A	151 ± 17.1 vs. 153 ± 15.1 *	N/A	0.198
		IL-17 (MFI)	336 ± 38.4 vs. 388 ± 34.8 *	N/A	396 ± 42.5 vs. 347 ± 30.3 *	N/A	0.206
		IFN-γ (MFI)	637 ± 54.9 vs. 558 ± 64.9 *	N/A	681 ± 55.6 vs. 624 ± 69.2 *	N/A	0.481
		TSH (μIU/mL)	3.7 ± 3.3 * vs. N/A	N/A	4.3 ± 7.1 * vs. N/A	N/A	N/A
		TPO-Ab (IU/mL)	258 ± 133 * vs. N/A	N/A	312 ± 123 * vs. N/A	N/A	N/A
		Tg-Ab (IU/mL)	551 ± 1094 * vs. N/A	N/A	396 ± 813 * vs. N/A	N/A	N/A
Robat-Jazi et al. [47]	50,000 (3 months)	25(OH)D (ng/mL)	25.3 ± 11.3 vs. 50.7 ± 15.3	N/A	19.9 ± 9.0 vs. 22.2 ± 9.7	N/A	<0.01
		IFN-γ (pg/mL)	13.9 ± 7.9 vs. 8.4 ± 4.8	0.001	14.1 ± 8.2 vs. 8.8 ± 5.6	<0.001	0.868
		TNF-α (pg/mL)	29.7 ± 18.4 vs. 15.3 ± 10.8	0.010	26.7 ± 24.8 vs. 12.3 ± 11.8	0.008	0.987
		TSH (μIU/mL)	3.7 ± 3.3 vs. N/A	N/A	4.3 ± 7.1 vs. N/A	N/A	N/A
		TPO-Ab (IU/mL)	258 ± 133 vs. N/A	N/A	312 ± 123 vs. N/A	N/A	N/A
		Tg-Ab (IU/mL)	551 ± 1094 vs. N/A	N/A	396 ± 813 vs. N/A	N/A	N/A

* mean ± SEM (standard error); TSH = thyroid-stimulating hormone; TPO-Ab =thyroid peroxidase antibodies; Tg-Ab = thyroglobulin antibodies; LT4 = L-thyroxine; 25(OH)D = 25-hydroxyvitamin D; CRP = C-reactive protein; IL-4 = interleukin 4; IL-10: interleukin 10; IL-17 = interleukin 17; IFN-γ = interferon gamma; TNF-α = tumour necrosis factor α; NS = not statistically significant; N/A = not detailed results available (information provided in the text of the paper).

**Table 3:** Selenium concentration vs. inflammatory, oxidative stress, antioxidant status, and thyroid parameters in an intervention and control group before and after selenium supplementation.

Authors	Selenium Dose and Form, μg/Day (Duration of Intervention)	Parameter (Unit)	Intervention Group Baseline vs. after Supplementation (Mean ± SD)	*p*-Value	Control Group Baseline vs. after Placebo/No Treatment (Mean ± SD)	*p*-Value	*p*-Value after Supplementationvs. Control
Chakrabarti et al. [40]	200, selenium acid (6 months)	MDA (mg/dL)	3.8 ± 2.0 vs. 1.8 ± 0.4	<0.001	4.3 ± 2.1 vs. 2.2 ± 0.5	<0.001	0.052
		TSH (μIU/mL)	25.8 ± 9.5 vs. 1.7 ± 0.7	<0.001	33.2 ± 23.5 vs. 1.7 ± 0.8	<0.001	N/A
		fT4 (ng/dL)	0.6 ± 0.1 vs. 1.6 ± 0.2	<0.001	0.6 ± 0.1 vs. 1.7 ± 0.2	<0.001	N/A
de Farias et al. [47]	200, selenomethionine (3 months)	Se (μg/L)	N/A vs. 63.4 ± 12.8	<0.001	N/A vs. 36.8 ± 9.6	NS	<0.001
		GPx1 (U/gHB)	58.4 ± 23.2 vs. 80.2 ± 12.1	<0.001	61.0 ± 21.7 vs. 61.8 ± 17.0	NS	<0.001
		TSH (μIU/mL)	1.7 ± 0.5 vs. 3.0 ± 1.5	<0.001	1.7 ± 0.4 vs. 2.2 ± 1.5	NS	N/A
		TPO-Ab (U/mL)	1009 ± 1015 vs. 958 ± 913 Δ: −5%	0.668	1206 ± 969 vs. 1405 ± 1070 Δ: 16%	N/A	N/A Δ:0.084
		Tg-Ab (U/mL)	510 ± 989 vs. 528 ± 997	N/A	521 ± 884 vs. 622 ± 1041	N/A	N/A
Esposito et al. [41]	166, selenomethionine (6 months)	CXCL10 (pg/mL)	N/A vs. N/A	NS	N/A vs. N/A	N/A	N/A
		TSH (μUI/mL)	2.7 ± 0.8 vs. N/A	NS	2.0 ± 0.4 vs. N/A	NS	NS
		fT3 (pmol/L)	N/A vs. ↑	<0.040	N/A vs. ↓	<0.02	N/A
		fT4 (pmol/L)	No change	NS	N/A vs. N/A	NS	N/A
		TPO-Ab (UI/mL)	2070 ± 575 vs. N/A	NS	3049 ± 757	NS	N/A
		Tg-Ab (N/A)	N/A vs. N/A	NS	N/A vs. N/A	NS	N/A
Hu et al. [29]	200, selenium yeast tablet (6 months)	Se (μg/L) (median)	73.6 vs. 187	<0.01	65.0 vs. 72.0	NS	<0.001
		SePP (ng/mL) (median)	16.0 vs. 17.2	<0.05	12.9 vs. 10.7	<0.01	0.007
		GPx3 (ng/mL) (median)	18.8 vs. 45.2	<0.01	18.5 vs. 24.2	<0.05	0.028
		TSH (mIU/L) (median)	3.2 vs. 2.4 Δ: −0.16	NS	2.8 vs. 3.2 Δ: 0.48	<0.01	0.021 Δ: 0.001
		fT3 (pmol/L)	4.4 ± 0.7 vs. 4.7 ± 0.7	NS	4.6 ± 0.7 vs. 4.6 ± 0.7	NS	0.691
		fT4 (pmol/L)	15.4 ± 2.6 vs. 16.4 ± 2.6	NS	15.7 ± 2.4 vs. 15.8 ± 1.9	NS	0.191
		TPO-Ab (IU/mL) (median)	237 vs. 178 Δ: −28.4	<0.01	177 vs. 211 Δ: 0	NS	0.942 Δ: 0.01
		Tg-Ab (IU/mL) (median)	435 vs. 388	NS	371 vs. 365	NS	0.891
Karanikas et al. [26]	200, sodium selenite (3 months)	Se (μg/L)	75 ± 11 vs. 125 ± 71	<0.05	76 ± 12 vs. 78 ± 12	NS	<0.05
		IL-2 (percentages)	CD4+ 67.3 ± 9.3 vs. 61.4 ± 10.7 CD8+ 30.6 ± 10.7 vs. 29.4 ± 14.9	NS NS	CD4+ 58.7 ± 13.6 vs. 64.6 ± 12.4 CD8+ 29.4 ± 14.3 vs. 29.4 ± 8.1	NS NS	NS NS
		IL-4 (percentages)	CD4+ 7.0 ± 3.2 vs. 7.1 ± 7.0 CD8+ 7.7 ± 9.3 vs. 5.0 ± 4.3	NS NS	CD4+ 6.6 ± 3.6 vs. 5.2 ± 2.6 CD8+ 6.9 ± 7.6 vs. 4.8 ± 5.1	NS NS	NS NS
		IL-10 (percentages)	CD4+ 6.9 ± 9.9 vs. 5.9 ± 7.9 CD8+ 1.2 ± 1.9 vs. 1.3 ± 2.6	NS NS	CD4+ 15.4 ± 26 vs. 5.8 ± 6.6 CD8+ 7.2 ± 27 vs. 2.2 ± 4.4	NS NS	NS NS
		IL-13 (percentages)	CD4+ 4.1 ± 1.7 vs. 5.1 ± 6.2 CD8+ 3.9 ± 3.4 vs. 3.3 ± 3.1	NS NS	CD4+ 4.6 ± 2.6 vs. 3.6 ± 1.8 CD8+ 5.7 ± 6.8 vs. 4.6 ± 6.6	NS NS	NS NS
		IFN-γ (percentages)	CD4+ 17.6 ± 8.6 vs. 17.9 ± 6.0 CD8+ 42.1 ± 20.6 vs. 38.7 ± 15.0	NS NS	CD4+ 18.7 ± 5.3 vs. 17.5 ± 8.7 CD8+ 42.0 ± 15.4 vs. 38.8 ± 17.9	NS NS	NS NS
		TNF-α (percentages)	CD4+ 67.4 ± 24.4 vs. 63.8 ± 24.0 CD8+ 48.6 ± 26.3 vs. 45.5 ± 25.7	NS NS	CD4+ 76.9 ± 11.7 vs. 68.3 ± 11.5 CD8+ 57.2 ± 16.7 vs. 48.2 ± 20.0	NS NS	NS NS
		TSH (μIU/mL)	2.1 ± 1.4 vs. 1.8 ± 0.8	NS	2.2 ± 1.7 vs. 2.0 ± 0.8	NS	NS
		fT4 (ng/dL)	1.5 ± 0.3 vs. 1.5 ± 0.4	NS	1.5 ± 0.4 vs. 1.5 ± 0.3	NS	NS
		TPO-Ab (IU/mL)	524 ± 452 vs. 505 ± 464	NS	521 ± 349 vs. 527 ± 354	NS	NS
Krysiak and Okopień [45]	200, selenomethionine (6 months) S: selenium LS: LT4 + Se L: LT4 P: placebo	hsCRP (mg/L)	S: 8.8 ± 1.5 vs. 4.4 ± 0.7LS: 8.5 ± 1.4 vs. 2.2 ± 0.4	<0.001 <0.001	L: 8.6 ± 1.2 vs. 4.3 ± 0.8 P: 8.0 ± 1.7 vs. 8.2 ± 1.8	<0.001 NS	S vs. *p* < 0.001 LS vs. P/L < 0.001 LS vs. S < 0.001 S vs. L NS L vs. P NS
		IL-1β (pg/mL)	S: 231 ± 34.2 vs. 188 ± 29.3 LS: 235 ± 29.0 vs. 103 ± 12.1	NS <0.001	L: 229 ± 23.2 vs. 140 ± 16.1 P: 220 ± 21.3 vs. 235 ± 30.1	<0.001 NS	L vs. S < 0.01 LS vs. P/L/S < 0.001 S vs. P NS L vs. P NS
		IL-2 (ng/mL)	S: 10.9 ± 1.5 vs. 6.8 ± 0.8 LS: 11.6 ± 1.9 vs. 4.9 ± 0.8	<0.001 <0.001	L: 11.4 ± 1.7 vs. 9.2 ± 1.6 P: 11.2 ± 1.3 vs. 10.8 ± 1.6	NS NS	S vs. *p* < 0.001 S vs. L < 0.05 LS vs. P/L < 0.001 LS vs. S < 0.01 L vs. P NS
		IL-6 (ng/mL)	S: 23.0 ± 2.8 vs. 19.0 ± 2.8 LS: 23.6 ± 2.5 vs. 11.9 ± 1.0	NS <0.001	L: 22.5 ± 2.3 vs. 14.4 ± 1.4 P: 22.9 ± 2.5 vs. 23.4 ± 2.9	<0.001 NS	L vs. S < 0.01 LS vs. P/S < 0.001 LS vs. L < 0.05 S vs. P NS L vs. P NS
		IFN-γ (ng/mL)	S: 133 ± 17.4 vs. 75.2 ± 9.5 LS: 129 ± 14.8 vs. 52.4 ± 7.4	<0.001 <0.001	L: 122 ± 14.6 vs. 103 ± 13.4 P: 126 ± 16.3 vs. 125 ± 20.4	NS NS	S vs. P/L < 0.001 LS vs. P/L/S < 0.001L vs. P NS
		TNF-α (pg/mL)	Monocyte: S: 2698 ± 312 vs. 2193 ± 198 LS: 2595 ± 348 vs. 1121 ± 135Lymphocyte: S: 792 ± 87 vs. 490 ± 51LS: 776 ± 79 vs. 349 ± 53	<0.001 <0.001 <0.001 <0.001	Monocyte: L: 2672 ± 321 vs. 1565 ± 186 P: 2560 ± 310 vs. 2598 ± 281 Lymphocyte: L: 803 ± 92 vs. 650 ± 65 P: 782 ± 82 vs. 762 ± 85	<0.001 NS NS NS	Monocyte: L vs. S < 0.001 LS vs. P/L/S < 0.001 S vs. L NS S vs. P NS Lymphocyte: S vs. *p* < 0.001 S vs. L < 0.01 LS vs. P/L/S < 0.001L vs. P NS
		MCP-1 (ng/mL)	S: 38.2 ± 4.2 vs. 30.6 ± 3.7 LS: 37.9 ± 4.4 vs. 18.6 ± 2.2	NS <0.001	L: 38.0 ± 4.6 vs. 23.2 ± 2.1 P: 37.2 ± 4.1 vs. 37.5 ± 3.9	<0.001 NS	L vs. S < 0.001 LS vs. P/S < 0.001 LS vs. L < 0.05 S vs. P NS L vs. P NS
Krysiak and Okopień [45]	200, selenomethionine (6 months) S: selenium LS: LT4 + Se L: LT4 P: placebo	TSH (mIU/L)	S: 2.18 ± 0.6 vs. 1.95 ± 0.6 LS: 2.27 ± 0.5 vs. 1.01 ± 0.3	NS <0.01	L: 2.24 ± 0.6 vs. 1.15 ± 0.4 P: 2.32 ± 0.6 vs. 2.21 ± 0.6	<0.001 NS	L vs. S < 0.05 LS vs. P/S < 0.001 LS vs. L NS S vs. P NS L vs. P NS
		fT3 (pmol/L)	S: 3.52 ± 0.28 vs. 3.75 ± 0.39 LS: 3.42 ± 0.34 vs. 4.46 ± 0.45	NS <0.001	L: 3.48 ± 0.35 vs. 4.39 ± 0.28 P: 3.46 ± 0.29 vs. 3.56 ± 0.35	<0.01 NS	LS vs. *p* < 0.01 S vs. LS/P/L NS LS vs. P NS L vs. P NS
		fT4 (pmol/L)	S: 14.3 ± 1.5 vs. 14.5 ± 1.3 LS: 13.9 ± 1.2 vs. 17.5 ± 1.5	NS <0.01	L: 14.1 ± 1.3 vs. 17.6 ± 1.7 P: 13.8 ± 1.2 vs. 14.3 ± 1.7	<0.01 NS	L vs. S < 0.05 LS vs. P/S < 0.05
		TPO-Ab (U/mL)	S: 1761 ± 375 vs. 1005 ± 331 LS: 1810 ± 452 vs. 463 ± 104	<0.01 <0.001	L: 1780 ± 328 vs. 1023 ± 294 P: 1723 ± 410 vs. 1884 ± 346	<0.01 NS	S vs. *p* < 0.001 LS vs. P/L/S < 0.001 S vs. L NS L vs. P NS
		Tg-Ab (U/mL)	S: 1565 ± 324 vs. 1312 ± 387 LS: 1695 ± 403 vs. 1291 ± 453	NS NS	L: 1650 ± 361 vs. 1320 ± 392 P: 1602 ± 308 vs. 1701 ± 355	NS NS	NS
Pilli et al. [44]	80 (I group) or 160 (II group), selenomethionine (12 months, 6th month, and 12th month assessment)	Se (μg/L) (median)	I: 84 vs. 112 (6th month, no further increase) II: 80 vs. 150 (6th month, no further increase)	<0.001 <0.001	82.1 vs. N/A	0.001	N/A
		SePP (mg/L)	no change	NS	N/A	N/A	N/A
		IFN-γ (pg/mL) (median)	baseline vs. 6th vs. 12th month: I: 9.7 vs. 7.3 vs. ↑ to baseline II: 8.9 vs. 7.8 vs. ↑ to baseline	6th month I: 0.017 II: 0.055 12th month I: NS II: NS	N/A	NS	N/A
		TNF-α (pg/mL)	baseline vs. 6th vs. 12th month: I: 12.4 vs. 9.9 vs. ↑ to baseline II: 12.1 vs. 10.1 vs. ↑ to baseline	6th month I: 0.016 II: 0.006 12th month I: NS II: NS	N/A	NS	N/A
		CXCL-9 (pg/mL) (median)	I: 70 vs. 40.9 (12th month) II: 66.4 vs. 49.0 (6th month, no further decrease)	I: 0.007 II: 0.001	N/A	0.012	<0.05
		CXCL-10 (pg/mL) median	I: 123 vs. 93.8 (12th month) II: 142 vs. 99.6 (12th month)	I: 0.017 II: 0.002	N/A	0.004	<0.05
		CXCL-11 (pg/mL) (median)	baseline vs. 6th vs. 12th month: I: 84.4 vs. 73.7 vs. ↑ to baseline II: 99.2 vs. 71.9 vs. ↑ to baseline	6th month I: 0.017 II: 0.001 12th month I: NS II: NS	N/A	NS	NS
Pilli et al. [44]	80 (I group) or 160 (II group), selenomethionine (12 months, 6th month, and 12th month assessment)	GPx3 (U/L)	no change	NS	N/A	N/A	N/A
		TSH (μU/mL)	baseline vs. 6th vs. 12th month: I: 2.4 ± 0.9 vs. N/A II: 2.6 ± 0.9 vs. N/A	N/A	2.2 ± 1.0	N/A	N/A
		fT3 (pg/mL)	baseline vs. 6th vs. 12th month: I: 3.1 ± 0.3 vs. N/A II: 3.1 ± 0.4 vs. N/A	N/A	3.2 ± 0.3	N/A	N/A
		fT4 (pg/mL)	baseline vs. 6th vs. 12th month: I: 8.2 ± 1.1 vs. N/A II: 8.1 ± 1.1 vs. N/A	N/A	8.3 ± 1.2	N/A	N/A
		TPO-Ab (U/mL) (median)	I: 410—stable II: 186—stable	NS	409 vs. 595 (6th month) vs. 518 (9th month) vs. stable	0.002 (9th month)	NS
		Tg-Ab (U/mL) (median)	I: no change II: 212 vs. 54.1	I: NS II: 0.007	144 vs. 87.8	0.0006	NS
		Thyroid volume (mL) (median)	baseline vs. 6th vs. 12th month: I: 10.5 vs. 9.7 vs. 9.6 II: 9.1 vs. 8.7 vs. 9.2	N/A	11.8 vs. 12.5 vs. 11.8	N/A	NS
		Thyroid echogenicity(gsp)	baseline vs. 6th vs. 12th month: I: 90.0 ± 20.4 vs. N/A II: 92.4 ± 14.9 vs. N/A	N/A	90.9 ± 13.1 vs. N/A	N/A	N/A
Preda et al. [46]	100, selenomethionine(3 months)	Se (μg/L)	258 ± 241 vs. 560 ± 363	0.001	237 ± 212 vs. 316 ± 160	0.014	0.001
		GPx1 (mU/dL)	0.64 ± 0.37 vs. 0.64 ± 0.38	0.979	N/A	N/A	N/A
		TSH (μUI/mL)	2.1 ± 1.0 vs. 2.5 ± 1.3	0.001	1.9 ± 1.1 vs. 2.4 ± 1.3	0.008	0.677
		TPO-Ab (UI/mL)	363 ± 348 vs. 307.9 ± 306.1	0.002	285 ± 235 vs. 290 ± 288	0.850	0.781
Sun et al. [32]	100, selenium yeast tablets (3 months)	IL-2 (N/A)	N/A vs. ↓	<0.05	N/A vs. ↓	NS	<0.05
		IL-10 (N/A)	N/A vs. ↑	<0.05	N/A vs. ↑	NS	<0.05
		TNF-α (N/A)	N/A vs. ↓	<0.05	N/A vs. ↓	NS	<0.05
		TSH (N/A)	N/A vs. ↓	<0.05	N/A vs. ↓	<0.05	NS
		TT3 (N/A)	N/A vs. ↑	<0.05	N/A vs. ↑	<0.05	NS
		TT4 (N/A)	N/A vs. ↑	<0.05	N/A vs. ↑	<0.05	NS
		TPO-Ab (N/A)	N/A vs. ↓	<0.05	N/A vs. ↓	<0.05	<0.05
		Tg-Ab (N/A)	N/A vs. ↓	<0.05	N/A vs. ↓	<0.05	<0.05
Tian et al. [34]	200, selenium yeast tablet (3 months)	Se (µg/L)	110 ± 16.3 vs. N/A	N/A	123 ± 19.1 vs. N/A	N/A	N/A
		MDA (nmol/mL)	6.8 ± 1.3 vs. 4.9 ± 0.7	<0.001	7.0 ± 1.6 vs. 7.2 ± 1.2	0.700	N/A
		TAC (mmol/L)	10.0 ± 1.9 vs. 12.9 ± 3.1	0.003	10.5 ± 2.5 vs. 9.2 ± 2.7	0.171	N/A
		SOD (U/mL)	72.3 ± 10.3 vs. 84.3 ± 13.2	0.007	69.1 ± 9.1 vs. 68.3 ± 11.4	0.832	N/A
		TSH (μUI/mL) median	1.7 vs. 1.4	0.734	1.94 vs. 2.12	0.935	N/A
		TPO-Ab (IU/mL) (median)	603 vs. 497	<0.001	581 vs. 569	0.110	N/A
		Tg-Ab (IU/mL) (median)	482 vs. 454	0.081	501 vs. 486	0.363	N/A
Wang et al. [35]	200 selenium yeast tablet (6 months) G1-S: subclinical AIT group with Se G1-P: subclinical AIT group with placeboG2-S: clinical AIT group with Se G2-P: clinical AIT group with placebo	Se (μg/L)	G1-S: 101 ± 19.3 vs. 181 ± 42.6 G2-S: 91.6 ± 19.9 vs. 188 ± 26.5	<0.01 <0.01	G1-P: 111 ± 27.9 vs. 106 ± 26.2 G2-P: 94.7 ± 21.1 vs. 87.1 ± 15.9	NS NS	G1-S vs. G1-*p* < 0.001 G2-S vs. G2-P < 0.001
		MDA (nmol/mL)	N/A vs. ↓	<0.001	N/A vs. ↑	0.026	NS
		GPx3 (U/mL)	N/A vs. ↑	<0.001	N/A vs. ↓	<0.001	NS
		TSH (mIU/L) (median)	G1-S: 3.3 vs. 3.2 G2-S: 2.1 vs. 2.5	NS NS	G1-P: 3.8 vs. 3.8 G2-P: 3.9 vs. 3.4	NS NS	G1-S vs. G1-P 0.318 G2-S vs. G2-P 0.219
		fT4 (pmol/L)	G1-S: 15.2 ± 3.1 vs. 14.1 ± 4.5 G2-S: 16.5 ± 2.4 vs. 15.3 ± 2.8	NS NS	G1-P: 14.7 ± 3.7 vs. 14.3 ± 4.3 G2-P: 15.3 ± 2.5 vs. 15.9 ± 3.9	NS NS	G1-S vs. G1-P 0.632G2-S vs. G2-P 0.922
		TPO-Ab (IU/mL) (median)	G1-S: 1020 vs. 873 Δ: −10.7% G2-S: 1310 vs. 960 Δ: −16.7%	<0.05 <0.05	G1-P: 977 vs. 930 Δ:−1.8% G2-P: 1037 vs. 1090 Δ: 1.2%	NS NS	G1-S vs. G1-P 0.643 Δ:0.105 G2-S vs. G2-P 0.845 Δ: 0.53
Yu et al. [36]	200 twice daily, selenium yeast tablet (3 months)	Se (μg/L) (median)	23.3 vs. 90.1 Δ:59.8	<0.05	24.0 vs. 39.6 Δ:8.2	NS	<0.001 Δ: <0.001
		Il-2 (pg/mL) (median)	216 vs. 159 Δ: −68.4	<0.05	212 vs. 227 Δ: −2.63	NS	<0.001 Δ: <0.001
		IL-10 (pg/mL) (median)	15.9 vs. 23.1 Δ: 7.7	<0.05	16.3 vs. 24.7 Δ: 7.3	<0.05	0.754 Δ: 0.80
		TPO-Ab (%)	33.6 ± 6.9 vs. 23.6 ± 9.3Δ: −10.0	<0.05	34.5 ± 8.6 vs. 32.0 ± 10.4 Δ: −2.5	NS	0.002 Δ: 0.002
		Tg-Ab (%)	50.4 ± 10.7 vs. 35.8 ± 15.2Δ: −14.6	<0.05	51.4 ± 11.5 vs. 45.5 ± 14.2 Δ: −6.0	NS	0.015 Δ: 0.011

AIT = autoimmune thyroiditis; TSH = thyroid-stimulating hormone; fT3 = free triiodothyronine; TT3 = total triiodothyronine; fT4 = free thyroxine; TT4 = total thyroxine; TPO-Ab = thyroid peroxidase antibodies; Tg-Ab = thyroglobulin antibodies; LT4 = L-thyroxine; hsCRP = high-sensitivity CRP; IL-1β = interleukin-1β; IL-2 = interleukin-2; IL-4 = interleukin-4; IL-6: interleukin 6; IL-10 = interleukin-10; IL-13 = interleukin-13; CD4+ = clusters of differentiation 4 positive; CD8+ = clusters of differentiation 8 positive; IFN-γ = interferon gamma; TNF-α = tumour necrosis factor α; MCP-1/CCL-2 = monocyte chemotactic protein-1/chemokine (C-C motif) ligand 2; CXCL-9 = C-X-C motif chemokine ligand 9; CXCL-10 = C-X-C motif chemokine ligand 10; CXCL-11 = C-X-C motif chemokine ligand 11; MDA = malondialdehyde; TAC = total antioxidant capacity; SOD = superoxide dismutase GPx1 = glutathione peroxidase 1; GPx3 = glutathione peroxidase 3; Se = selenium; SePP = selenoprotein P; NS = not statistically significant; N/A = not detailed results available (information provided in the text of the paper); Δ: difference after supplementation/placebo vs. baseline values; ↓ = decrease in levels/activity; ↑ = increase in levels/activity.

**Table 4:** Inflammatory status, oxidative stress, antioxidant status, and thyroid parameters in an intervention and control group before and after DS with ingredients other than vitamin D or selenium.

Authors	Supplement, Form, Dose (Duration of Intervention)	Parameter (Unit)	Intervention Group Baseline vs. after Supplementation (Mean ± SD)	*p*-Value	Control Group Baseline vs. after Placebo (Mean ± SD)	*p*-Value	*p*-Value after Supplementationvs. Control
Rabbani et al. [30]	30 mg zinc gluconate/day 250 mg magnesium oxide/day 25,000 IU vitamin A twice a week (10 weeks)	hsCRP (mg/dL) (median)	3.2 vs. 3.0 Δ: −0.13	0.007	2.9 vs. 3.2 Δ: 0.18	0.004	0.154 Δ: <0.001
		MDA (mg/dL) (median)	16.7 vs. 12.3 Δ: −1.60	0.408	11.0 vs. 12.6 Δ: 0.03	0.182	0.666 Δ: 0.119
		TAC (mg/dL) (median)	2.2 vs. 2.2 Δ: 0.03	0.378	2.4 vs. 2.1 Δ: −0.14	0.004	0.364 Δ: 0.007
		TSH (mIU/L) (median)	1.6 vs. 1.2 Δ: −0.08	0.966	1.1 vs. 1.4 Δ: −0.02	0.319	0.601 Δ: 0.308
		fT3 (pg/mL) (median)	3.0 vs. 3.1 Δ: 0.07	0.293	3.3 vs. 3.2 Δ: 0.10	0.984	0.179 Δ: 0.473
		fT4 (ng/dL) (median)	1.2 vs. 1.4 Δ: 0.12	0.002	1.3 vs. 1.2 Δ: 0.00	0.618	0.034 Δ: 0.007
		TT4 (μg/dL) (median)	6.6 vs. 6.7 Δ: 0.20	0.165	6.5 vs. 6.8 Δ: 0.00	0.856	0.531 Δ: 0.434
Nordio and Basciani [42]	Wobenzym vital (4 tablets twice a day for 1 week, 2 tablets twice a day for the rest of the 6 month period) (6 months) W: Wobenzym W + LT4: Wobenzym + LT4 (without placebo; LT4 in the control group)	CRP (mg/L)	W: 6.3 ± 1.9 vs. 2.9 ± 1.3 W + LT4: 6.7 ± 1.4 vs. 2.2 ± 1.2	<0.05 <0.05	5.8 ± 2.8 vs. 3.1 ± 1.8	NS	N/A
		TSH (mcIU/mL)	W: 3.3 ± 1.4 vs. 2.6 ± 1.2 W + LT4: 3.8 ± 1.0 vs. 0.7 ± 03	NS <0.05	3.6 ± 1.1 vs. 1.2 ± 0.4	NS	N/A
		fT3 (N/A)	N/A vs. ↓	NS	N/A vs. ↓	NS	N/A
		TPO-Ab (IU/mL)	W: 1017 ± 674 vs. 619 ± 473 W + LT4: 937 ± 518 vs. 475 ± 327	NS NS	971 ± 574 vs. 818 ± 379	NS	N/A
		HTg-Ab (IU/mL)	W: 896 ± 312 vs. 435 ± 323 W + LT4: 814 ± 242 vs. 387 ± 168	NS <0.05	787 ± 298 vs. 662 ± 304	NS	N/A
		HTg (ng/mL)	W: 37.6 ± 16.1 vs. 16.4 ± 9.3 W + LT4: 38.8 ± 13.9 vs. 14.5 ± 10.7	<0.05 <0.05	41.1 ± 11.7 vs. 36.6 ± 12.3	NS	N/A
Farhangi and Tajmiri [28]	2 g/day *Nigella sativa* powder (8 weeks)	MDA (nmol/mL)	1.7 ± 0.7 vs. 1.4 ± 0.4	0.034	1.7 ± 0.7 vs. 1.6 ± 0.6	0.53	0.53
		TAC (nmol/l)	1.3 ± 0.3 vs. 1.4 ± 0.3	0.04	1.1 ± 0.4 vs. 1.2 ± 0.3	0.182	0.41
		SOD (IU/L)	1286 ± 351 vs. 1390 ± 282	0.05	1251 ± 980 vs. 1211 ± 357	0.98	0.67
		GPx (IU/L)	45.1 ± 17.9 vs. 47.0 ± 10.6	0.56	39.2 ± 18.1 vs. 40.6 ± 3.8	0.67	0.61
		TSH (mIU/L)	6.4 ± 3.9 vs. 4.1 ± 2.4	0.03	8.1 ± 7.3 vs. 8.3 ± 7.2	0.40	0.02
		T3 (mmol/L)	0.9 ± 0.3 vs. 1.1 ± 0.3	0.008	1.2 ± 0.4 vs. 1.2 ± 0.4	0.15	0.39
		T4 (mmol/L)	8.1 ± 2.6 vs. 8.9 ± 1.4	0.21	8.0 ± 3.1 vs. 7.7 ± 2.2	0.32	0.04
		TPO-Ab (IU/mL)	295 ± 210 vs. 148 ± 158	0.019	278 ± 171 vs. 274 ± 167	0.28	0.01
Zhang et al. [3]	600 mg/day genistein (purified soy extract) (1 month)	IL-2 (pg/mL)	11.8 ± 2.2 vs. 7.5 ± 1.9	<0.01	N/A	NS	N/A
		IL-4 (pg/mL)	N/A	NS	N/A	NS	N/A
		IL-6 (pg/mL)	N/A	NS	N/A	NS	N/A
		IL-10 (pg/mL)	N/A	NS	N/A	NS	N/A
		IFN-γ (ng/mL)	12.0 vs. 16.4	<0.05	N/A	NS	N/A
		TNF-α (pg/mL)	no change	NS	N/A	NS	N/A
Zhang et al. [3]	600 mg/day genistein (purified soy extract) (1 month)	TSH (mU/L)	12.8 ± 3.1 vs. 8.8 ± 2.3	<0.01	N/A	N/A	N/A
		T3 (ng/mL)	112 ± 26.5 vs. 131 ± 25.3	NS	N/A	N/A	N/A
		T4 (μg/dL)	9.5 ± 2.5 vs. 12.7 ± 2.7	<0.05	N/A	N/A	N/A
		fT4 (μg/dL)	0.9 ± 0.2 vs. 1.3 ± 0.3	<0.01	N/A	N/A	N/A
		TPO-Ab (U/mL)	1108 ± 239 vs. 789 ± 173	<0.01	N/A	N/A	N/A
		Tg-Ab (U/mL)	764 ± 152 vs. 436 ± 146	<0.01	N/A	N/A	N/A
Tomella et al. [42]	Fermented papaya-based nutraceutical, 3 g twice a day (6 months)	MDA (μmol/L)	N/A vs. 0.3 ± 0.3	NS	N/A vs. 0.7 ± 0.2	<0.01	<0.05
		L-HPX (nmol/L)	N/A vs. 3.1 ± 0.8	NS	N/A vs. 6.2 ± 0.6	<0.01	<0.05
		SOD (U/L)	N/A vs. 30.8 ± 2.6	NS	N/A vs. 23.6 ± 2.2	NS	<0.05
		GPx (U/L)	N/A vs. 649 ± 94.3	NS	N/A vs. 689 ± 102	<0.01	<0.05
Talebi et al. [32]	Familact (synbiotic) 500 mg/day: (8 weeks)	CRP (mg/dL)	1.4 ± 0.5 vs. 2.5 ± 0.9 Δ: 0.58	0.006	2.5 ± 1.0 vs. 1.6 ± 0.3 Δ: 0.34	0.250	0.699
		TSH (μUI/mL)	2.0 ± 0.3 vs. 1.4 ± 0.2 Δ: −0.28	0.007	1.5 ± 0.2 vs. 1.3 ± 0.2 Δ: −0.08	0.358	0.374
		fT3 (pg/mL)	2.4 ± 0.1 vs. 2.8 ± 0.1 Δ: 0.38	0.001	2.3 ± 0.1 vs. 2.7 ± 0.04 Δ: 0.35	0.001	0.259
		TPO-Ab (IU/mL)	220 ± 38.9 vs. 233 ± 40.5 Δ: 13.6	0.161	118 ± 36.4 vs. 121 ± 36.2 Δ: 2.78	0.157	0.317
Xiang et al. [26]	Alpha lipoic acid 300 mg/day (3 weeks)	CRP (mg/L)	2.9 ± 0.4 vs. 2.5 ± 0.5	NS	2.7 ± 0.4 vs. 2.8 ± 0.4	NS	N/A
		TSH (mU/L)	9.3 ± 2.8 vs. 10.2 ± 2.8	NS	10.5 ± 2.5 vs. 11.1 ± 2.5	NS	N/A
		fT3 (pmol/L)	5.1 ± 0.6 vs. 5.2 ± 0.7	NS	5.0 ± 0.7 vs. 5.2 ± 0.6	NS	N/A
		fT4 (pmol/L)	14.0 ± 2.1 vs. 14.8 ± 2.0	NS	15.0 ± 2.2 vs. 14.9 ± 2.6	NS	N/A
		TPO-Ab (U/mL)	542 ± 287 vs. 582 ± 295	NS	506 ± 268 vs. 496 ± 298	NS	N/A
		Tg-Ab (U/mL)	465 ± 306 vs. 487 ± 289	NS	492 ± 311 vs. 473 ± 295	NS	N/A

Wob = Wobenzym (mixture of plant-based enzymes, bioflavonoids, vitamin C, D, and E); Familact = seven freeze-dried probiotic strains (7 × 10^9^ colony forming units: (CFU) Lactobacillus Casei, 2 × 10^9^ CFU Lactobacillus Acidophilus, 1.5 × 10^9^ CFU Lactobacillus Rhamnosus, 2 × 10^8^ CFU Lactobacillus Bulgaricus, 2 × 10^10^ CFU Bifidobacterium Breve, 7 × 10^9^ CFU Bifidobacterium Longum, 1.5 × 10^10^ CFU Streptococcus Thermophilus), fructooligosaccharide as a prebiotic; TSH = thyroid-stimulating hormone; fT3 = free triiodothyronine; T3 = triiodothyronine; fT4 = free thyroxine; T4 = thyroxine; TT4 = total thyroxine; TPO-Ab = thyroid peroxidase antibodies; Tg-Ab = thyroglobulin antibodies; HTG = High-sensitivity human thyroglobulin; HTg-Ab = High-sensitivity human thyroglobulin antibodies; LT4 = L-thyroxine; hsCRP = high-sensitivity CRP; IL-1β = interleukin-1β; IL-2 = interleukin-2; IL-4 = interleukin-4; IL-6: interleukin 6; IL-10 = interleukin-10; IFN-γ = interferon gamma; TNF-α = tumour necrosis factor α MDA = malondialdehyde; GPx = glutathione peroxidase; TAC = total antioxidant capacity; SOD = superoxide dismutase; L-HPX = plasma hemopexin; NS = not statistically significant; N/A = not detailed results available (information provided in the text of the paper); Δ: difference after supplementation/placebo vs. baseline values; ↓ = decrease in levels/activity; ↑ = increase in levels/activity.

## Data Availability

Not applicable.

## Supplementary Materials

The following supporting information can be downloaded at: https://www.mdpi.com/article/10.3390/antiox12101798/s1, Table S1: Literature searching strategy, Table S2: Results of the quality assessment of randomised studies using the CASP Randomised Controlled Trial Standard Checklist, Table S3: Results of the quality assessment of non-randomised studies with ROBINS-I.
