# Supplementary material for: Do Dietary Supplements Affect Inflammation, Oxidative Stress, and Antioxidant Status in Adults with Hypothyroidism or Hashimoto’s Disease?—A Systematic Review of Controlled Trials
Source: Antioxidants (Basel). 2023 Sep 24;12(10):1798. doi: 10.3390/antiox12101798 (PMC10604871; doi:10.3390/antiox12101798)
Supplement: Supplementary file 1 [file antioxidants-12-01798-s001.zip › antioxidants-2611413-supplementary.pdf]

Table S1. Literature searching strategy

Search number	Search term	PubMed	Scopus	Web of Science
#1	(hypothyroid) OR (hypothyroidism) OR (autoimmune thyroiditis) OR (Hashimoto's)	106,155	104,997	49,124
#2	(vitamin) OR (mineral) OR (vitamin A) OR (retinol) OR (carotenoid) OR (beta-carotene) OR (β-carotene) OR (vitamin B1) OR (thiamine) OR (vitamin B2) OR (riboflavin) OR (vitamin B3) OR (niacin) OR (nicotinic acid) OR (nicotinamide) OR (vitamin B5) OR (pantothenic acid) OR (vitamin B6) OR (pyridoxine) OR (pyridoxal) OR (pyridoxamine) OR (vitamin B7) OR (biotin) OR (vitamin B9) OR (folate) OR (folic acid) OR (vitamin B12) OR (cobalamin) OR (B-complex) OR (B complex) OR (vitamin C) OR (ascorbic acid) OR (vitamin D) OR (cholecalciferol) OR (vitamin E) OR (alpha-tocopherol) OR (α-tocopherol) OR (vitamin K) OR (phylloquinone) OR (menaquinones) OR (multivitamin) OR (calcium) OR (magnesium) OR (iron) OR (zinc) OR (iodine) OR (selenium) OR (multimineral) OR (dietary supplements) OR (supplementation) OR (omega-3) OR (probiotic) OR (symbiotic) OR (myo-inositol) OR (inositol) OR (coenzyme Q10) OR (alpha lipoic acid) OR (DHA) OR (docosahexaenoic acid) OR (EPA) OR (eicosapentaenoic acid)	2,538,234	5,530,956	3,626,179
#3	(inflammation) OR (oxidative stress) OR (DNA damage) OR (antioxidant capacity) OR (antioxidant status) OR (albumin) OR (interleukine) OR (NADPH oxidase) OR (leptin) OR (proinflammatory cytokines) OR (cytokine) OR (ROS) OR (reactive oxygen species) OR (adipocytokine) OR (serum inflammatory markers) OR (CRP) OR (C reactive protein) OR (neutrophil/lymphocyte ratio) OR (tumor necrosis factor-α) OR (tumor necrosis factor) OR (TNF-a) OR (TNF) OR (interleukin-6) OR (IL-6) OR (interleukin-12) OR (IL-12) OR (interleukin-4) OR (IL-4) OR (8-epi-PGF2a) OR (hsCRP) OR (interleukin-10) OR (IL-10) OR (interleukin-8) OR (IL-8) OR (interleukin-13) OR (IL-13) OR (interleukin-2) OR (IL-2) OR (interleukine-1B) OR (IL-1B) OR (interleukine-5) OR (IL-5) OR (interleukine-17) OR (IL-17) OR (interferon gamma) OR (IFN) OR (lipid peroxidation) OR (protein oxidation) OR (catalase) OR (glutathione peroxidase) OR (GPx) OR (glutathione reductase) OR (GR) OR (SOD) OR (superoxide dismutase) OR (isoprostanes) OR (F2-isoprostanes) OR (F2-IsoPs) OR (neuroprostanes) OR (protein carbonyl) OR (MDA) OR (malondialdehyde) OR (8-hydroxydeoxyguanosine) OR (8-OHdG)	3,123,163	3,424,621	2,658,335
#4	#1 AND #2 AND #3	1827	3004	911

Table S2. Results of the quality assessment of randomised studies using the CASP Randomised Controlled Trial Standard Checklist [23] - Sections A and B

Authors, year	Section A: Is the basic study design valid for a randomised controlled trial?			Section B: Was the study methodologically sound?				
	Did the study address a clearly focused research question?	Was the assignment of participants to interventions randomised?	Were all participants who entered the study accounted for at its conclusion?	Were the participants 'blind' to intervention they were given?	Were the investigators 'blind' to the intervention they were giving to participants?	Were the people assessing/analysing outcome/s 'blinded'?	Were the study groups similar at the start of the randomised controlled trial?	Apart from the experimental intervention, did each study group receive the same level of care (that is, were they treated equally)?
Anaraki et al., 2017	Yes	Yes	Yes	Yes	Yes	Can't tell	Yes	Yes
de Farias et al., 2015	Yes	Yes	No	Yes	Yes	Can't tell	Yes	Yes
Esposito et al., 2017	Yes	Yes	Can't tell	Yes	Yes	Can't tell	Yes	Yes
Farhangi & Tajmiri, 2020	Yes	Yes	Yes	Yes	Yes	Can't tell	Yes	Yes
Hu et al., 2021	Yes	Yes	Can't tell	No	No	Can't tell	Yes	Yes
Karanikas et al., 2008	Yes	Yes	Can't tell	Yes	No	No	Yes	Yes
Krysiak & Okopień, 2011	Yes	Yes	Yes	Yes	Yes	Yes	Yes	Yes
Nodehi et al., 2019	Yes	Yes	Yes	Yes	Yes	Can't tell	Yes	Yes
Pilli et al., 2015	Yes	Yes	Can't tell	Yes	Can't tell	Can't tell	Yes	Yes
Preda et al., 2017	Yes	Yes	Yes	Can't tell	Can't tell	Can't tell	Yes	Can't tell
Rabbani et al., 2021	Yes	Yes	Yes	Yes	Yes	Can't tell	Yes	Can't tell
Robat-Jazi et al., 2022	Yes	Yes	Can't tell	Yes	Yes	Can't tell	Yes	Can't tell
Sun et al., 2021	Yes	Yes	No	Can't tell	Can't tell	Can't tell	Yes	No
Talebi et al., 2020	Yes	Yes	Yes	Yes	Yes	Yes	Yes	Yes
Tian et al., 2020	Yes	Yes	Yes	Can't tell	Can't tell	Can't tell	Yes	Yes
Wang et al., 2018	Yes	Yes	No	Yes	Yes	Can't tell	Yes	No
Xiang et al., 2010	Yes	Yes	Yes	Can't tell	Can't tell	Can't tell	Yes	No
Yu et al., 2017	Yes	Yes	Yes	Yes	Yes	Can't tell	Yes	Yes
Zhang et al., 2017	Yes	Yes	No	Yes	Yes	Can't tell	Yes	Yes

Table S2. *Continuation* - Sections C and D

Authors, year	Section C: What are the results?			Section D: Will the results help locally?		**Overall assessment**
	Were the effects of intervention reported comprehensively?	Was the precision of the estimate of the intervention or treatment effect reported?	Do the benefits of the experimental intervention outweigh the harms and costs?	Can the results be applied to your local population/in your context?	Would the experimental intervention provide greater value to the people in your care than any of the existing interventions?	
Anaraki et al., 2017	No	Can't tell	No	No	Can't tell	Major limitations
de Farias et al., 2015	Yes	Yes	Can't tell	No	Can't tell	Major limitations
Esposito et al., 2017	Can't tell	Can't tell	Can't tell	Can't tell	Can't tell	**Minor limitations**
Farhangi & Tajmiri, 2020	Yes	Can't tell	Yes	Yes	Can't tell	**Minor limitations**
Hu et al., 2021	Can't tell	Yes	Yes	No	Can't tell	Major limitations
Karanikas et al., 2008	No	No	Yes	Yes	Can't tell	Major limitations
Krysiak & Okopień, 2011	Can't tell	Yes	Can't tell	Yes	Can't tell	**Minor limitations**
Nodehi et al., 2019	Can't tell	Yes	No	No	Can't tell	Major limitations
Pilli et al., 2015	Can't tell	Yes	No	Yes	Can't tell	Major limitations
Preda et al., 2017	No	Can't tell	Yes	Can't tell	Can't tell	Major limitations
Rabbani et al., 2021	Yes	Yes	Yes	Can't tell	Can't tell	**Minor limitations**
Robat-Jazi et al., 2022	No	Can't tell	No	No	Can't tell	Major limitations
Sun et al., 2021	No	Can't tell	No	No	Can't tell	Major limitations
Talebi et al., 2020	Yes	Yes	Can't tell	No	Can't tell	Major limitations
Tian et al., 2020	Can't tell	Yes	No	Can't tell	Can't tell	Major limitations
Wang et al., 2018	Can't tell	Yes	Yes	Can't tell	Can't tell	Major limitations
Xiang et al., 2010	Can't tell	Can't tell	Can't tell	Can't tell	Can't tell	**Minor limitations**
Yu et al., 2017	Can't tell	Yes	Yes	Can't tell	Can't tell	**Minor limitations**
Zhang et al., 2017	Can't tell	Can't tell	Can't tell	Can't tell	Can't tell	Major limitations

Table S3. Results of the of the quality assessment of non-randomised studies with ROBINS-I [25]

Bias domain	Signalling questions	Chakrabarti et al., 2016	Nordio & Basciani, 2015	Tomella et al., 2014
Bias due to confounding	1.1 Is there potential for confounding of the effect of intervention in this study? If N/PN to 1.1: the study can be considered to be at low risk of bias due to confounding and no further signalling questions need be considered.	No	No	No
	1.4. Did the authors use an appropriate analysis method that controlled for all the important confounding domains?	Yes	No information	Probably yes
	1.5. If Y/PY to 1.4: Were confounding domains that were controlled for measured validly and reliably by the variables available in this study?	Yes	No information	Probably yes
	1.6. Did the authors control for any post-intervention variables that could have been affected by the intervention?	No	No information	No
	1.7. Did the authors use an appropriate analysis method that controlled for all the important confounding domains and for time-varying confounding?	Probably yes	Probably yes	Yes
	1.8. If Y/PY to 1.7: Were confounding domains that were controlled for measured validly and reliably by the variables available in this study?	Yes	Probably yes	Probably yes
Risk of bias judgement		Low	Low	Low
Bias in selection of participants into the study	2.1. Was selection of participants into the study (or into the analysis) based on participant characteristics observed after the start of intervention?	No	No	No
	2.4. Do start of follow-up and start of intervention coincide for most participants?	Yes	Yes	Yes
Risk of bias judgement		Low	Low	Low
Bias in classification of interventions	3.1 Were intervention groups clearly defined?	Yes	Yes	Yes
	3.2 Was the information used to define intervention groups recorded at the start of the intervention?	Yes	Yes	Yes
	3.3 Could classification of intervention status have been affected by knowledge of the outcome or risk of the outcome?	No	No information	No information
Risk of bias judgement		Low	Low	Low
Bias due to deviations from intended interventions	4.1. Were there deviations from the intended intervention beyond what would be expected in usual practice?	No	No	No
Risk of bias judgement		Low	Low	Low
Bias due to missing data	5.1 Were outcome data available for all, or nearly all, participants?	Yes	Yes	No information
	5.2 Were participants excluded due to missing data on intervention status?	No	No	No information
	5.3 Were participants excluded due to missing data on other variables needed for the analysis?	No	No	No information
	5.4 If PN/N to 5.1, or Y/PY to 5.2 or 5.3: Are the proportion of participants and reasons for missing data similar across interventions?	-	-	No information
	5.5 If PN/N to 5.1, or Y/PY to 5.2 or 5.3: Is there evidence that results were robust to the presence of missing data?	-	-	No information
Risk of bias judgement		Low	Low	No information
Bias in measurement of outcomes	6.1 Could the outcome measure have been influenced by knowledge of the intervention received?	Yes	Probably yes	Probably yes

	6.2 Were outcome assessors aware of the intervention received by study participants?	Probably yes	Yes	Probably yes
	6.3 Were the methods of outcome assessment comparable across intervention groups?	Yes	Yes	Yes
	6.4 Were any systematic errors in measurement of the outcome related to intervention received?	No information	No information	No information
Risk of bias judgement		Moderate	Moderate	Moderate
Bias in selection of the reported result	Is the reported effect estimate likely to be selected, on the basis of the results, from...			
	7.1. ... multiple outcome measurements within the outcome domain?	No	No	Probably no
	7.2 ... multiple analyses of the intervention-outcome relationship?	No	No	No
	7.3 ... different subgroups?	No	No	No
Risk of bias judgement		Low	Low	Low
**Overall bias**	**Risk of bias judgement**	**Moderate**	**Moderate**	**Moderate**
